# Covidom, a Telesurveillance Solution for Home Monitoring Patients With COVID-19

**DOI:** 10.2196/20748

**Published:** 2020-10-22

**Authors:** Youri Yordanov, Agnes Dechartres, Xavier Lescure, Caroline Apra, Pascaline Villie, Jerome Marchand-Arvier, Erwan Debuc, Aurélien Dinh, Patrick Jourdain, On Behalf Of The AP-HP / Universities / Inserm COVID-19 Research Collaboration

**Affiliations:** 1 Sorbonne Université, AP-HP, Hôpital Saint Antoine, Service d'Accueil des Urgences, INSERM, Institut Pierre Louis d'Epidémiologie et de Santé Publique, UMR-S 1136 Paris France; 2 Sorbonne Université, INSERM, Institut Pierre Louis d'Epidémiologie et de Santé Publique, UMR-S 1136, AP-HP, Hôpital Pitié Salpêtrière, Département de Santé Publique, Centre de Pharmacoépidémiologie de l’AP-HP (Cephepi) Paris France; 3 Department of Infectious and Tropical Diseases Bichat-Claude Bernard University Hospital Assistance Publique-Hôpitaux de Paris Paris France; 4 Service de Neurochirurgie Hôpital Pitié Salpêtrière Assistance Publique-Hôpitaux de Paris – Sorbonne Université Paris France; 5 Assistance Publique-Hôpitaux de Paris Paris France; 6 Service d'Accueil des Urgences Hôpital Saint Antoine Assistance Publique-Hôpitaux de Paris – Sorbonne Université Paris France; 7 Infectious Disease Department R Poincaré University Hospital, Garches Assistance Publique-Hôpitaux de Paris – Paris Saclay University Paris France; 8 Université Paris Saclay, AP-HP, Hopital du Kremlin Bicêtre, Service de Cardiologie, Département médico-universitaire Coeur Vaisseaux Reins, Kremlin Bicêtre, France Kremlin-Bicetre France; 9 AP-HP / Universities / Inserm COVID-19 Research Collaboration Paris France

**Keywords:** COVID-19, coronavirus disease, home monitoring, telesurveillance, monitoring, patient, infectious disease, app

## Abstract

In a matter of months, COVID-19 has escalated from a cluster of cases in Wuhan, China, to a global pandemic. As the number of patients with COVID-19 grew, solutions for the home monitoring of infected patients became critical. This viewpoint presents a telesurveillance solution—Covidom—deployed in the greater Paris area to monitor patients with COVID-19 in their homes. The system was rapidly developed and is being used on a large scale with more than 65,000 registered patients to date. The Covidom solution combines an easy-to-use and free web application for patients (through which patients fill out short questionnaires on their health status) with a regional control center that monitors and manages alerts (triggered by questionnaire responses) from patients whose health may be deteriorating. This innovative solution could alleviate the burden of health care professionals and systems while allowing for rapid response when patients trigger an alert.

## Introduction

In less than 7 months, COVID-19 escalated from a cluster of cases in Wuhan, China, to a global pandemic with more than 15 million infected people and 630,000 deaths in over 200 countries [1,2]. The clinical characteristics of patients with COVID-19 are well described, with most presenting mild symptoms and fatalities occurring mainly in chronically ill and older patients [3-7]. In addition to being a therapeutic challenge for physicians and health care workers, the exponential increase in patients with COVID-19, and their considerable length of stay in a hospital, could exceed health care systems’ capacities [8-12]. To allow hospitals to focus on vulnerable and the most severely ill patients, those with COVID-19 but presenting no serious symptoms are being sent home [13]. However, for 10% to 15% of these patients, the disease will become severe, which requires surveillance [13,14]. Various systems have been set up to carry out this surveillance, often involving general practitioners (GPs) and telephone-based and/or home visits, or the use of telehealth technologies for virtual consultations [15,16]. However, all these systems rely on the individual management of every patient by a single doctor (GP, infectious disease specialist, or any other specialist involved in COVID-19 management). In a pandemic situation, GPs and infectious disease specialists are scarce resources and should be mobilized wisely [15,17,18].

To offer alternatives to patients while reserving medical resources for the situations that require it, the Greater Paris University Hospitals (Assistance Publique-Hôpitaux de Paris, [APHP]), in collaboration with regional GP organizations and a software company specializing in patient digital pathways, quickly developed a remote telesurveillance solution named Covidom for the home monitoring of patients with COVID-19.

## The Covidom Solution

**Figure 1:**
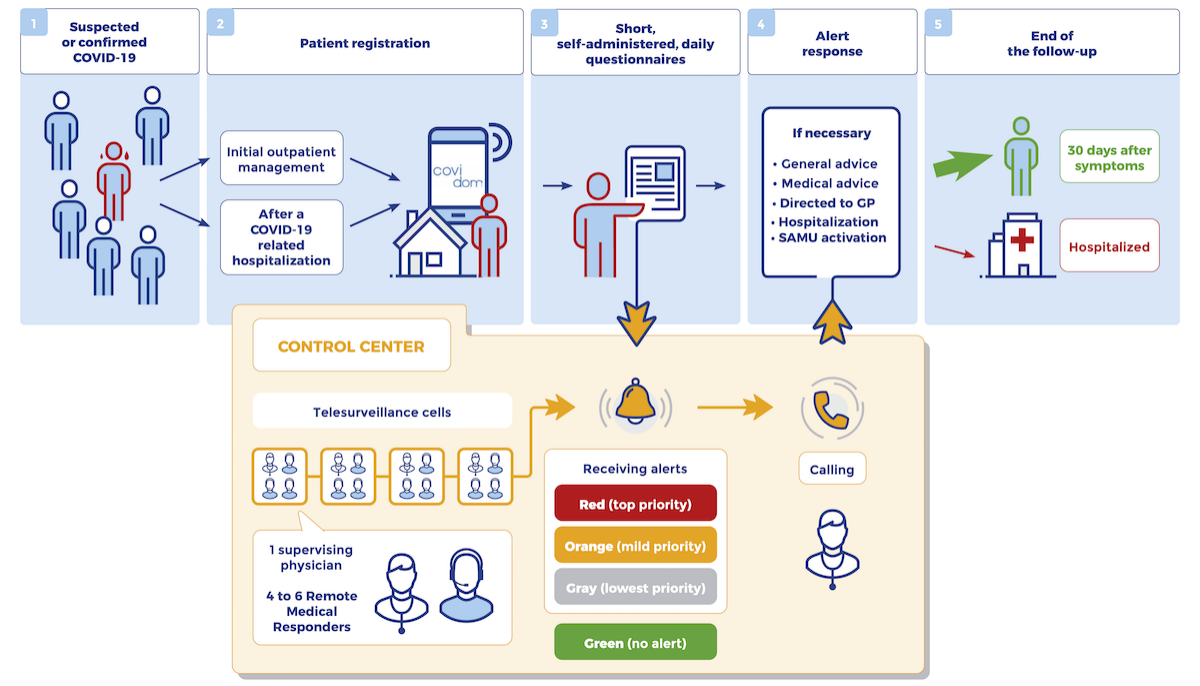
The Covidom solution: patient pathway and regional control center organization. GP: general practitioner, SAMU: Service d’Aide Médicale Urgente.

Covidom combines an easy-to-use and free web application for patients with a regional control center to manage alerts (Figure 1). Patients with a suspected or confirmed case of COVID-19, according to the French public health authorities’ definition of COVID-19 infection [19], are registered by a physician after receiving a brief description of the Covidom solution and providing oral consent to participate. Registration can be performed either as part of outpatient management after diagnosis (ie, after a visit to an emergency department or consultation with a GP or another specialist) or at the time of discharge after COVID-19–related hospitalization. Registration is a simple procedure where patients provide simple baseline characteristics, including age, gender, phone number or email address, date of first symptoms, and risk profile. A high-risk profile includes the presence of cardiovascular disease, diabetes, chronic lung disease, immunodeficiency (transplant, active cancer treatment, uncontrolled HIV infection, etc), third trimester of pregnancy, or age >65 years [19].

Patients then receive a registration link via a short mobile message or email, through which they complete registration and provide electronic consent for the Covidom telesurveillance program. They are informed of the potential use of their anonymized data for research purposes. This use was approved by the scientific and ethical committee of APHP (IRB00011591).

The data is available upon request for academic researchers.

### The Covidom Web Application

**Figure 2:**
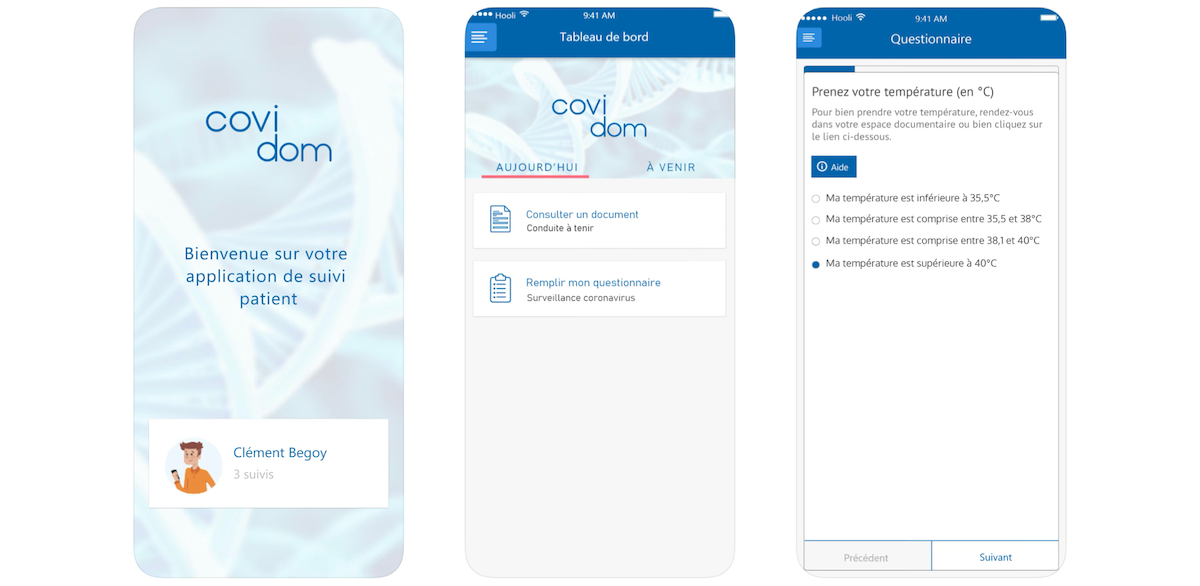
Covidom web application screenshots.

The web application was designed to be straightforward and intuitive to use for patients. The interface of the application can be seen in Figure 2. Patients complete one or two self-administered daily monitoring questionnaires for a duration of 30 days after symptom onset. These questionnaires involve fewer than 10 short and standardized questions. The questionnaires can be accessed via computer or smartphone, and patients are informed by mobile message or email to complete them with a reminder in case of no response. The questions ask patients to self-report their respiratory rate, heart rate, temperature, respiratory uneasiness (adapted from the modified Borg dyspnea scale [20]), nausea, malaise, as well as psychological discomfort and difficulties dealing with lockdown measures. Patients assessed as high risk by the physician who performed their initial evaluation need to complete the questionnaires twice a day, while low-risk patients respond only once a day. The answers to these monitoring questionnaires can trigger different types of alerts at the regional control center. These questionnaires were elaborated and tested by a panel of multidisciplinary health care professionals (infectious diseases, emergency physicians, GPs, and telesurveillance specialists).

In the web application, patients can also find information on the virus and how to mitigate transmission risk (ie, French health ministry documents); how to measure one’s own temperature, heart rate, and respiratory rate; and how to seek psychological support if needed. In case of an emergency, patients are advised to directly contact the national emergency number by dialing 15 (Service d’Aide Médicale Urgente [SAMU]).

### The Covidom Regional Control Center

The Covidom regional control center is open from 8 AM to 8 PM, 7 days a week, and covers all patients using the Covidom system in the greater Paris area (12 million inhabitants). It is built on the concept of autonomous remote monitoring cells. Each cell is made up of 4 to 6 trained remote monitoring responders (RMRs) and a supervising physician, all physically colocated at the Covidom regional control center, equipped with face masks and adhering to physical distancing measures. The mission of the cells is to handle the alerts generated by patient answers to the daily or twice-daily questionnaires. Patient answers are classified into 4 categories by an automated algorithm:

- No alert: everything is considered normal, no need for further action;
- Orange alert: some of the answers are above a certain threshold. These alerts need a response from the regional control center;
- Red alert: some of the answers suggest that the patient’s condition may be deteriorating. These alerts need a response from the control center, with the highest level of priority;
- Gray alert: the patients did not answer the questionnaire. These alerts need a response from the control center and patients need to be called, but the level of priority is low.

To handle the alerts, RMRs access the patient record and contact patients by phone to identify the cause of the alert. If needed, the supervising physician of the cell can intervene and assess the patient over the phone. An alert is considered handled once the RMR or the physician offers a solution to the patient: general advice, medical advice, directed to their GP, hospitalization, or contact with the SAMU. Infectious disease wards, emergency departments, or the SAMU can be contacted directly by the control center using dedicated phone numbers. If necessary, these contacts could result in a mobile intensive care unit staffed with emergency physicians sent to the patient’s home or a regular ambulance with a paramedic sent to assess the patient and transport them to a hospital. Of note, in the case of remote medical assessment, Covidom personnel does not charge a fee.

The control center cell physicians and RMRs are volunteers from different backgrounds (Multimedia Appendix 1). Physicians are rarely infectious disease specialists, GPs, or emergency physicians since those individuals are on the frontline caring for patients in need of acute care. Covidom personnel are mostly other specialists with decreased activity because of the lockdown who wanted to contribute to crisis management. All physicians and RMRs receive theoretical and practical training, the intensity of which depends on the person’s profile. They do not receive any financial incentives, but nonfinancial incentives are offered, such as meals or transportation solutions if public transport is not available. All volunteers have to sign an individual contract with the APHP for medical confidentiality, insurance, and liability reasons. On-site psychological support is available if needed.

## Overview of Covidom as of May 19, 2020

In the period between March 9 to May 19, 2020, 57,182 patients were registered in Covidom with a suspected or confirmed case of COVID-19. These patients were referred by 1709 physicians working in 30 public and 70 private hospitals and by 2131 GPs (in private or public medical practices). Most patients were referred as part of their initial outpatient care (50,020/57,182, 87.5%) while 7162 were included at hospitalization discharge. Out of these patients, 84.5% (48,290/57,182) confirmed their registration, 8.4% (4057/48,290) never answered a surveillance questionnaire, and 70.6% (34,104/48,290) answered questionnaires for more than 7 days. A total of 104 patients were offered alternatives, by contacting patients’ GPs to organize a follow-up, as they had trouble using the system (eg, uncomfortable using the required technologies or language issues). Patients’ mean age was 43.7 years (SD 15.8) and a majority were female (33,542/48,290, 58.7%) (Table 1). The patients’ risk profile was recorded as high in 60.3% (3315/5493) of cases included at hospital discharge and in 39.9% (17,082/42,797) of cases included as part of their initial outpatient care.

**Figure 3:**
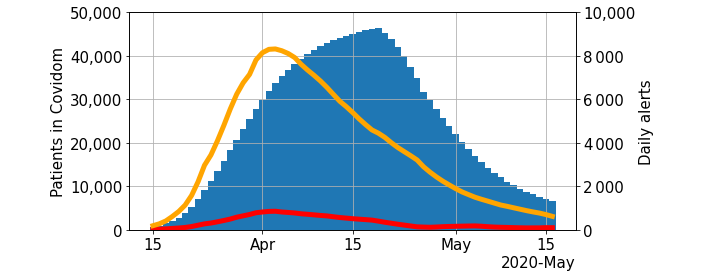
Number of patients and alerts over time.

During follow-up (phases 3 and 4 of the patient’s pathway in Figure 1), patients triggered 21,873 red alerts and 211,160 orange alerts. Red alerts were handled in a median time of 2 min and 20 s (IQR 46 s to 6 min and 54 s) and orange alerts were handled in 10 min and 34 s (IQR 1 min and 28 s to 93 min and 51 s). We present in Figure 3 the weekly averaged counts of patients managed using Covidom and the alerts generated. From March 30, 368 alerts resulted in contact with SAMU (via the national emergency number) through the regional control center (over 215,056 alerts by 41,758 patients). As of May 19, 72.0% (34,767/48,290) of patients had their follow-up terminated, 1.1% (544/48,290) had been hospitalized or rehospitalized, and 0.1% had died (39/48,290).

**Table 1:** General characteristics of and reasons for end of follow-up among patients using Covidom, as of May 19, 2020.

Characteristic			Posthospital discharge management (n=5493)	Initial outpatient management (n=42,797)	Total (N=48,290)
**General characteristics**					
	Age (years), mean (SD)		48.5 (17.2)	42.3 (14.9)	43.7 (15.8)
	**Gender, n (%)**				
		Male	2669 (48.6)	16,260 (38.0)	23,564 (41.2)
		Female	2818 (51.3)	26,488 (61.9)	33,542 (58.7)
**Risk profile, n (%)**					
	High-risk profile		3315 (60.3)	17,082 (39.9)	24,756 (43.3)
**Reason for end of follow-up, n (%)**					
	Automatic termination of follow-up after 30 days		3957 (72.0)	30,810 (72.0)	34,767 (72. 0)
	Follow-up ended early at patient’s request^a^		831 (15.1)	7473 (17.5)	8304 (17.2)
	Ongoing follow-up		590 (10.74)	4046 (9.45)	4636 (9.60)
	Hospital admission		111 (2.0)	433 (1.0)	544 (1.1)
	Death		4 (0.1)	35 (0.1)	39 (0.1)

^a^Follow-up ended early at patient’s request: no more symptoms, no longer felt like answering questionnaires, or any other reason left at the patient’s discretion.

## Implications, Future Works, and Limitations

To our knowledge, Covidom is the first and largest telesurveillance solution (65,202 patients as of July 24) described for the home monitoring of COVID-19 cases with the aim to alleviate the burden of health care professionals and systems. Telesurveillance has never been used in acute infectious diseases at this scale until now [21]; previously, it had been mostly used in chronic diseases [22,23]. Most health care systems are based on in-person interactions between patients and their clinicians, but in a pandemic context, this situation is highly challenged, and health digital solutions are of interest both to patients and to the health care system [15-17]. From patients’ perspective, this system can provide reassurance by daily monitoring their condition with a procedure in place in case of worsening symptoms. Patients often worry about the potential and sudden worsening of symptoms during a lockdown, due to limited social contact [24,25]. This telesurveillance system allows for close but minimally invasive surveillance using daily short questionnaires with fewer than 10 questions, which is easily accepted by patients [26]. From the public health perspective, this system may offer a partial virtual safety net to rapidly detect any signs of deterioration in patients with COVID-19, while making proper use of scarce resources via a 2-step process where automated alert algorithms can trigger a medical response when needed. Automatic algorithms and health care professionals based in a regional control center could help reserve health care workers and hospital beds for the patients who need them most and alleviate pressure on the health care system.

Such tools also have the major advantage of ensuring close surveillance while avoiding physical contact, which can help limit the spread of the virus and possible health care worker contamination. Providing appropriate care while preserving one’s own health is a strong motivation for health care workers to rapidly develop and widely use virtual health care solutions [27].

Finally, Covidom represents an important source of epidemiologic data, providing an opportunity to increase our knowledge of the disease, in particular of its common but least studied mild form. Covidom was initially deployed in the greater Paris area but is being extended to other French regions using the same principles: use of a web application with a dedicated regional control center whose functioning may depend on the region. Because of its simplicity and quick response, this solution could be easily adapted in other countries.

Of course, the Covidom solution needs to be thoroughly evaluated; in particular, the efficiency and ability of the alerts to detect patients at high risk of deterioration and the medico-economic impact of such a solution need evaluation. To do this, we will link the Covidom database with hospitals and national social security databases to identify patients who directly contacted them or were self-referred to an emergency department.

The Covidom system was sustainable during the lockdown due to the personnel availability that resulted from nonurgent elective procedures or appointments being rescheduled; most of the workforce comprised salaried employees (as opposed to a pay-per-service system). We observed significant fluctuations in the availability of human resources. At first, and due to the lockdown, many volunteers offered their help. Since lockdown measures were lifted (May 11, 2020) and as control center cell physicians and RMRs progressively resumed their usual activities, finding enough personnel has become more of a challenge. Adapting the Covidom solution and offering alternatives to patients who had difficulty with the system (unfamiliar with these technologies or language issues) was done by connecting these patients to their GPs. However, additional options could have included translated versions of the questionnaires and the other available documents to help patients with language issues. Surveillance could also have been more flexible as it was found to be short for some patients with recurrent or persistent symptoms, while others would have preferred to stop the follow-up as soon as the symptoms disappeared. Finally, sharing the patients’ Covidom file, or a summary of it, with patients’ GPs was not done.

## Conclusion

Covidom is an innovative solution for the home monitoring of patients with COVID-19. The model could be easily transposed to other countries or contexts. Most patients have been included as part of their initial outpatient management, making Covidom the largest cohort to date of patients with a mild case of COVID-19, which is the form that occurs in a majority of patients but is least studied. Using telesurveillance solutions like Covidom could augment health care systems’ abilities by allowing them to monitor patients and promptly identify worsening symptoms, while limiting the need to travel and the risk of contamination.

## Appendix

Multimedia Appendix 1 List of physicians, supervising physicians, and remote monitoring responders part of the Covidom system.

## Abbreviations

APHP: Assistance Publique-Hôpitaux de Paris
GP: general practitioner
RMR: remote monitoring responder
SAMU: Service d’Aide Médicale Urgente
URPS: Union régionale des professionnels de santé
