# Supplementary material for: Covidom, a Telesurveillance Solution for Home Monitoring Patients With COVID-19
Source: J Med Internet Res. 2020 Oct 22;22(10):e20748. doi: 10.2196/20748 (PMC7644373; doi:10.2196/20748)
Supplement: Multimedia Appendix 1 [file jmir_v22i10e20748_app1.doc]

**Appendix 1.** Covidom including physicians, supervising physicians and remote monitoring responders

*Supervising physicians and remote monitoring responders :* ABAD CAROLE, ABAKKA Samya, ABDESSEMED Yassir, ABEN DANAN Shaoul, ABI KHALIL Mathilde, ABITBOL Camille, ABITBOL Samuel, ABOU TAAM Rola, ABRAMOWITZ Laurent, ABREU Romain, ABRUNHOSA MADEIRA Alisson, ACHIR maya, ACHOUR Ilana, ACKERMANN Elina, ADAMSBAUM Catherine, ADER FLAVIE, ADHAM Salma, ADOUANI Hana, AFENJAR alexandra, AGIER Alexis, AGNETTI Richard, AGRANAT Pascal, AGUT Richard, AH-FAT Robin, AITTERRAMI brahim, AL-REFAI Maissoun, ALAIE alexandre, ALBER Anna, ALBERTINI Caroline, ALBERTUS Claire-Marine, ALBISETTI Amélie, ALEXANDRE Clémence, ALEXANDRE-HEYMANN Laure, ALEXANDROV Lukas, ALIMI GHARIANI Anne Sophie, ALLAIN Margaux, ALLAINGUILLAUME Amandine, ALLALI Yann, ALLEAUME SOPHIE, ALOVA Ilona, ALSIOUFI MOHAMAD LEO, AMADOR Maria del Mar, AMALOTPAVATHAS PRIANGA, AMAR Matteo, AMARA Barka, AMARA Mounia, AMAT Elisabeth, AMAT FLORE, AMAZZOUGH karima, AMELOT Mathilde, AMERIOU Laura, AMEZIANE Annissa, AMIACH Léa , AMIEL HELENE, AMOUYAL Chloé, AMOUZGAR Mardjan, AMSELLEM Eden, AMSLER Emmanuelle, AMUSAN Jean etienne, AMZALLAG Léa, AMZALLAG Micheline, ANDRES PASCAL, ANNEREAU Jean-Philippe, ANTOINE Ambre, ANTOINE DUVERNOIS REGINE, ANTOUN HADI, AOUNI Dalil, APRA Caroline, APTÉ Claire, ARCHAMBEAUD Marie-Pierre, ARCIDIACONO Giulia, ARGAND Erwan, ARMAGAN Alexandre, ARMILHAC DEZAMIS ALEXANDRA, ARNAUD Laurie , ARNOULD Clemence, ARQUES Florence, ARTIGOU catherine, ARTIGOU Hélène, ARTIGOU Jean-Yves, ASCHEHOUG isabelle, ASMANE-DE LA PORTE IRENE, ASSABAH Hicham, ASSALIT Ines, ASSARAF Sylvia laura, ASSELINEAU ALAIN, ASSIER Haudrey, ASSOULINE Benjamin, ASSOUS Sam, ATASSI-DUMONT Marie-Elisabeth, ATLAN Michael, ATTAL Laurence, ATTAL Patrick, ATTAL STYM POPPER Sarah, ATTALI Pierre, ATTHAR Léa, ATTICOT Julie, AUBER elise, AUBOURG Frédérique, AUBRY Alexandra, AUDARD Virginie, AUDAT Francoise, AUGER Florence, AUGER Manon, AURIAU Johanne, AUSSILHOU beatrice, AUTIN Maia, AVOUAC Jérôme, AYACHI Samy, AYMARD Nicolas, AZEDE Amandine, AZOULAY-CAYLA Arièle, BA Souleymane, BAAKILI Adyb, BAAROUN vanessa, BABA AISSA Idriss, BABADJIAN Célia, BACHELOT Guillaume, BADER-MEUNIER brigitte, BADOZ Emma, BAH Garanké, BAH Rabiatou, BAILLIEU Marie, BAILLIF Christophe, BAJARD Pierre, BAJOLLE FANNY, BALAIRE Victoria, BALOGH ROCHER Christina, BALTAGI Elias, BANNEVILLE beatrice, BARBANT Chloé, BARBIER Antoine, BARBOTEAU Clément, BARBU Charlotte, BARNIER Jean-Philippe, BARON Marguerite, BARONNET Veronique, BARRAUD-LANGE Virginie, BARRAULT LAURE, BARRAUX Océane, BARRE marie, BARRY Djenabou, BARTAGNON Hugo, BARTOLI Alexandra, BATEL Leila, BATISSE Anne, BAUCHARD Arthur, BAUDRIER Pierre, BAUMANN MOREL clarisse, BAYCE-CHALVIN Clémence, BAYE Dina, BAYON DE NOYER Alix, BEAUFRERE Marie, BEAUJARD thomas, BEAUMONT Pascale, BEAUMONT Quitterie, BECQUEMONT laurent, BEDIAT Audrey, BEGON BAGDASSARIAN Isabelle, BEGOS Maïda, BEIGNEUX Ysoline, BEKEL Lilia, BELAHOUEL Mehdi, BELENUS Nicolas, BELIN Adèle , BELKACEMI Anastasia, BELLAICHE MICCIO Annie, BELLESME Céline, BELLITY Rachel, BELLON Marie, BELLON Nathalia, BELO Sephora, BELOT-GUERY MARTINE, BELOUNIS Rachida, BEN AMOR Farès, BEN JABALLAH Hamdi, BEN YAOU rabah, BEN YOUNES-UZAN carine, BENAÏSSA Nihad, BENAMAR Yasmine, BENARROCH Yaël, BENASSAIA Laurence, BENATTAR Laura, BENAYOUN Raphael, BENDAVID Alexandre, BENDERRA Marc-Antoine, BENGUIGUI Jonas, BENGUIGUI-HERSAN Maxime, BENITAH Malkiel, BENIZRI Chloé, BENMAGHNIA Sofiane , BENMEZIANE Fatima, BENOIST Alexandra, BENOIST stephane, BENOMAR Dalia, BENOSMAN Hedi, BENSAADI Yacine, BENSALAH Amel, BENSIMHON Jessica, BENSOUSSAN Louise, BENUSIGLIO Patrick, BERDAH Yoel, BERENBAUM Jules, BERESSI NATHALIE, BERGERON Margaux, BERLIN Ivan, BERNARD SOLDEVILA Marion, BERNIER Sophie, BERROU Vinciane, BERSTEL-DA SILVA Martine, BERTEIN Nadège, BERTERETCHE Marie-Violaine , BERTHAT Virginie, BERTHO BAPTISTE, BESNARD Clémentine, BESSALAH HOUCINE, BESSE Cécile, BEST Anne-Laurence, BEUGRE GBANE ROKIA SANDRINE, BEYDON Nicole, BEYLER Constance, BEZIER Cassandre, BEZILLE Marie, BIARD Coraline, BIDARRA Léana, BIEHLER Pascal, BIETH Theophile, BIHAN Kevin, BILLON Clarisse, BINET DECAMPS VERONIQUE, BIOUTI Inès, BIRET Edwin, BISDORFF BRESSON Annouk, BISSETTE Eva, BITAR Aimee, BITTON Shana, BLAISE Romain, BLANC Angélique, BLANCHARD Christine, BLANCHARD Cyril, BLANCHET Benoit, BLARD Maxence, BLOHM Nathalie, BLOM Sabine, BLUMENTAL Yaël, BOCCARA Olivia, BOCCARD Victorine, BOCSKEI Eszter, BODILIS Helene, BOËDEC Corinne, BOHL Jean-Baptiste, BOIGE NATHALIE, BOISSONNAS Alain, BOLGERT Francis, BONALDI Alice, BONAVITA Adrien, BONEU valerie, BONFILS Naureen, BONIN Noëlle, BONNEFOY annick, BONNEFOY Arnaud, BONNET Anne-Laure, BONNET Isabelle, BONNET joelle, BONNET Lina, BONNETAIN Mathilde, BONNIN Sophie, BONNY Lison, BONTURI jc, BORDARIER Cecile, BORDEAUX Sixtine, BORDEN Alaina, BORDET céline, BORDET Emilie, BORIES Pauline, BORNENS Pia, BOROCCO Charlotte, BOTAS Salomé, BOTTOIS Cecile, BOUABID Fadia, BOUAIN Sarra, BOUANANI Ines, BOUCHER Alyzée, BOUCHER LUCIE, BOUCHERON Pauline, BOUCHET Hugo, BOUEDEC. CHADENIER Gaëlle, BOUGARD-DELLA SIGNORA AMANDINE, BOUGNOUX Marie-Elisabeth, BOUHASSIRA Didier, BOUHIER éric, BOUHLAL Inès, BOUILLERET Viviane, BOUJENAH Rebecca, BOUJU LE TAILLANDIER Valerie, BOUKRAA Emir, BOULANOUAR Miriam, BOULAS Mathieu, BOULAY Noémi, BOULIC-MAILLARD Pauline, BOULLET Mary, BOUMLOUGUA Soleima, BOUN Justin, BOURDON Coralie, BOURGOIN Alice, BOURGOIN Cécile, BOUSCHON Mathilde, BOUSQUET Guillemette, BOUTIN Antoine, BOUTY Marie, BOUYGUES Emma, BOUZOUITA Imen, BOUZY Juliette, BOYER Antoine , BOYER CHAMMARD Timothée, BRACHET Virginie, BRAHIMI Hanane, BRAMBILLA Sandrine, BRANCHEREAU Sophie, BRANCHET Eric, BRANDEL Jean-Philippe, BRANELLEC albanne, BRANELLEC albanne, BRASSIER ANAIS, BRASSIER Julia, BRAULT Emilie, BRESSON Angélique, BRETON SYLVIE, BREUIL Laetitia , BRIAND AUDREY, BRIATTE Inès, BRICAIRE François, BRICAIRE LEOPOLDINE, BRISSET Claire, BROCKER Laurent, BROUARD Agnès, BROUARD Marie, BROUARD Rémi, BRUGMAN Thomas, BRUN Adrian, BRUN Thibaud, BRUNELLE Julie, BUFFET Alexandre, BUFFET PIERRE, BULIN Noémie, BULTEZ Maëllanne, BUNEL Simon, BUNGE Lucie, BURASCHI Helene, BURIN DES ROZIERS Cyril, BURY Thierry, BUSQUET philippe, BUSQUEU Agnes, BUSSON Emmanuel, BUTEAU FABRE Cyrielle, CACACE Cyril, CALLE Mélissa, CALMEL Vincent, CALVEZ Aude, CALVO Anne-Sophie, CAMHAJI Nicolas, CAMPAGNE Victor, CAMPEDEL Luca, CAMPERGUE mathilde, CAMPOS César, CAMPOY Faustine, CAMUS-BABLON Florence, CANALE sandra, CANTALLOUBE Alberte, CAPLAN Kevin, CARCAUZON Gilles, CARETTE Anne, CARLES PONCE Laura, CARNEIRO RODRIGUES Ilidia, CARRIE Alain, CARTE--JACQUESSON Julien, CARTIER Natacha, CARVIGAN Jim, CASTAGNA Julie, CASTAN Pierre-Antoine, CASTELOT Ilona , CASTIEL Philippe, CATALA Annaelle, CATTAN ELIE, CAUSSIN Elisa, CAVALERIE Jean-Lin, CAZENOVES Alexandre, CEDRIN Isabelle, CEKOVIC Dorian, CERF-BENSUSSAN Nadine, CERVERA Pascale, CHABBERT-BUFFET Nathalie, CHALLIER Jordan, CHAMBRIN LAUVRAY Hélène, CHAMBRIN Pauline, CHAMPALOUX Bernard, CHAMPEAU William, CHANEAC JOBARD Aline, CHANTEBEL Léa, CHANTELOT Corentin, CHAOUACHI Leila, CHAPELON EMELINE, CHAPIRO Elise, CHAPOUTIER Caroline, CHARLES Adeline, CHARLES Perrine, CHARLIER Lucile, CHARLOT Maryse, CHARLOT Maxime, CHARLOT-PONSART Anne, CHARPENTIER marie-christine, CHARPY cécile, CHARRON Philippe, CHARTON Valentine, CHASSAING Caroline, CHASTEL BOURGUET Anne, CHATEAU Alexis, CHATEAUNEUF Christine, CHATELAIN Sarah, CHAUVEAU Justine, CHAUVEAU-TISSIER Anne-Marie, CHAUVEL Clémentine, CHAZE Anna, CHECROUN Valerie, CHEDOUBA leila, CHENA SAMIA, CHENET Flore, CHENUET Mathis, CHERIFI Katia, CHESNEAU Jonathan, CHESNEL Camille, CHEVALIER Anne sophie, CHEVALIER Emmanuelle, CHEVALIER Julia, CHEVALIER Valérie, CHEVANCE Virgile, CHIADMI Mohammed Fouad, CHIBANE Nesrine, CHIKHI Sara, CHOQUET Sylvain, CHOUCHANA Laurent, CHOUGAR Taous, CHOURAQUI Nawel, CHRISTOZOVA Viliana, CHRYSSOSTALIS Ariane, CHUNG Sui-Sheng, CIMBERT Shaza, CISSE Marieme, CITOUNADIN Isadora, CITTERIO helene, CLABASSI Lara, CLARICO Melvil, CLAUSER Sylvain, CLEMENT annick, CLEMENT Gaetan, CLEMENT Valentine, CLERAMBAULT Audrey, CLERGEOT Alain, CLOUARD Lucile, COBAN Axelle, COBELAS Lorraine, COCHARD Baptiste, COCHENNEC Laure, COHEN Shani, COHEN SOLAL Eric, COHEN Sophie, COIGNARD eva, COLAS DES FRANCS Claire, COLAS DES FRANCS véronique, COLAS Pascale, COLIN ALEXANDRE, COLIN Amandine, COLIN DE VERDIERE NATHALIE, COLLIER Laure, COLLIGNON Anne-Margaux , COLLIOT Isabelle, COMBOURG Marine, COMMEAU Cloé, CONÇU Thibault, CONGARD-CHASSOL Brigitte, CONSOLI Angele, CONSTANS DIANE, CONTOUR Zélie , CORDOLIANI Florence, CORIAT Pierre, CORNIC Delphine, CORRE Matthieu, CORSAND Félix, CORTE Helène, COSTA Clément, COSTANTINO Félicie, COSTE-ZEITOUN delphine, COTTEL Nathalie, COTTET Virginie, COUCKE Philippe, COUET Sebastien, COULEUR Laurine, COULON Justin, COURBEBAISSE MARIE, COURCIER Soizic, COUREUX Françoise, COURTAULT-DESLANDES Flora, COURTIN Thomas, COURTINAT Félicie, COURTOIS-DE LA CHAPELLE Clémence , COUSINOU Elodie, COUVERT Philippe, COUVIN Delphine, COVALI Ala, COYER francoise, COZIC Jean Philippe, CREPELLE Sylvie, CRETOLLE Célia, CREUSOT Soline, CRICKX Béatrice, CURAN Jean, CURRAS Arthur, CUSSAC Laure-Anne, CUSSENOT ISABELLE, CYRILLE Stacy, D'HARCOURT Blaise, DA COSTA Hugo, DA SILVA VIEIRA Dylan, DABOUT Anaëlle, DACHERY Pierre, DAELMAN Chantal, DAGRENAT Céline, DAHAN Candice, DAHAN Sandrine, DAHEL Daryn, DAHMANI Medi, DAILLAND Philippe, DALIBARD Florian, DALLANT Titiane, DALMAT Olivia, DAMIANO Maria, DANG Catherine, DANGLES Marie-Thérèse, DANJOU Isabelle, DANNEELS Sandrine , DANON-LAURENT LAURENCE, DANTAGNAN Claire-Adeline, DANZIGER Nicolas, DAOUD CREMA Michel, DAOUDI Aurore, DARNIEAUD Audrey, DARVISH Elsa, DAUBERTON Aurore, DAUDE Orianne, DAUTZENBERG bertrand, DAUVERGNE Eva, DAVY Gauthier, DAY Nesrine, DAYRAS Aurélie, DE BASTARD Arthur, DE BASTARD Laurent, DE BELENET Claire, DE BOISSIEU Delphine, DE BURETEL DE CHASSEY Flore, DE COUESSIN Anne-Charlotte, DE FOURMESTRAUX Pierre, DE GENNES christian, DE LANNOY Lucy, DE LAROCHE Marine, DE LONLAY Pascale, DE LUCIA Silvana, DE MILLY Marie-Noëlle, DE SAISSET Charles, DE SILVA Christine, DE SOUSA Ana sofia, DE VERGNES Nathalie, DEBIEN Patricia, DEBRAS Elodie, DEBRAY Dominique, DEBRE timothe, DEBS RABAB, DECEUNINCK Claire, DECQUE Karen, DEFLANDRE Didier, DEFLANDRE Jean-Michel, DEGENNE Remi, DEGONDE Julie, DEGUINE Romain, DEL GENES Elodie, DELAGRAVE Elisabeth, DELAHAYE-DURIEZ Andrée, DELALANDRE Coline, DELAVAL Maelle, DELAVAL MOLKO Agnès, DELAYE Clémence, DELBE-BERTIN Laëtitia, DELCOUR Clémence, DELEAGE Martine, DELESPINE MARIE-HELENE, DELETTE Anne, DELIERE Odile, DELILLE Chloé, DELILLE Marie-Antoinette, DELTEIL Florence, DEMANGE Hugo, DENEBOUDE David, DENEUX Catherine, DENIS Agathe, DENIS Céline, DENIS Martine, DENJOY ISABELLE, DEPAGNE Gilles, DERACINOIS DRESS Céline, DERICBOURG Coraline, DERIEUX Julia, DEROUEN julie, DERRAS Rahma, DERUELLE Clémentine, DESBOEUF Stephane, DESBORDES Camille, DESCAMPS Marouchka, DESCHAMPS Claire, DESCORPS DECLERE Julien, DESMOUSSEAUX Marine, DESNOS Michel, DESPLAN Saïna, DESTEXHE Zoé, DESTREZ CATHERINE, DEVILLE Marie, DEVILLE RACHEL, DEWERDT Dominique, DHEILLY Anthony, DIAVOLO Melina, DICKO michele, DIEZ-JAHIER Gwenaelle, DILLE Lucie, DINIS Clorie, DION elisabeth, DIOT Geneviève , DJABOUR Alizé, DJADOUN BAYA, DO ESPIRITO SANTO Julien, DODE HARDELIN Catherine, DODIN Louise, DOLO Léonie, DOMERGUE Alizée, DOMINJON Fabienne, DOMITILLE de Mascureau, DONADILLE Bruno, DONDELINGER Eloïse, DOREL Valérie, DORFMANN Marc, DORIA julia, DOUAOURIA Sophia, DOUCHY Laura, DOUMET Lynn, DOURNES Aveline, DOURTHE Marie Emili, DOUSSOT Raphael , DRAY Nathalie, DREYFUS-SCHMIDT Elsa, DROUELLE Sylvie, DROUHET Thibault, DROUINEAUD Véronique, DROZE Jessica, DRUCKER Corinne, DRUYLANS Eva, DU VIGNAUX Alix de, DUBERGEY Julie, DUBERN Beatrice, DUBOIS Marie, DUBOS Laura, DUBOURG genevieve, DUC Philippe, DUCASTEL Guillaume, DUCHMANN Matthieu, DUCLOUX Françoise, DUDAS Thi HONG hanh, DUHAMEL Louis, DULIEU Albane, DUMAS Sophie, DUMAS Vincent, DUME Florie, DUMEIGE Laurence, DUMILLARD Céline, DUMONT Maëlle, DUOLE Simon, DUONG Tu Anh, DUPONT Edouard, DUPONT Jean-Michel, DUPONT Patricia, DUPONT Sylvie, DUPRE Clément, DUPUIS philippe, DUPUY Tiphaine, DURAND Emmanuel, DURAND Marie-Christine, DURAND Pascale, DURCHON Chloé, DURE Laurène, DURIEUX Alexandra, DUSSAULE Claire, DUSSAULE Jean-Claude, DUSSER Anais, DUTHEIL PASCALE, DUTOUR Léa, DUVAL Anna, DUVERNE Louis, DUVERT Aurélie, DWERNICKI Zoé, DZIUBICH Marie dominique, ECALE Clotilde, EGEE Pauline, EHLINGER jeremie, EID Camille, EID Joseph, EL AMRAOUI Zephyr, EL KAIM Audrey, EL KHOURY Lara, EL MOUHADI BARNIER Sanaa, EL SAKHAWI Karim, EL SISSY Carine, ELMENSI ridha, ELOY Gauthier, ENGELMANN-CHARNEAU Marjolaine, ENICOLO Claire, EPAUD CHRISTELLE, EPELBOIN Sylvie, EPSTEIN MADELEINE, ESKENAZI Sarah, ESNAULT GELLY PASCALE, ETCHEMENDY Ilona, ÉTIENNE Léo, EUVE Tatiana, EVIN Cecile, EWENCZYK Claire, EYRAUD Thomas, EYSSETTE Stéphanie, EZAOUI Simon, FABIEN Marie, FACY Marie, FAILLAUFAIX JULIE, FAILLOT thierry, FAIVRE Lionel, FAJAC Isabelle, FAMILY Delphine, FARCET Anaïs, FARIS Imane, FARWATI Haani, FAUDET ANNE, FAUTREL Arnaud, FAUTREL Bruno, FAVIN-LEVEQUE Domitille, FEGHALI Rose, FEKI Kenza, FEL Audrey, FELTIN Louise, FENAIN Laurène, FENISTEIN Myriam, FERAUX Anaelle, FERDOWS Edwin, FERNANDES CARVALHO Philippe, FERNET AYMERIC, FERRAND Eve, FERRAND-JUNG Thibaut, FERTEY Juliette, FERY Elisabeth, FEUVRET Loic, FEYRIT Louison, FICHES Catherine, FIDAHOUSSEN Sarah, FILSER Mathilde, FIMATIS Carole-Anne, FINELLE Laurent, FINKIELSZTEJN Laurent, FISCHER Evelyne, FLAMBARD Laeticia, FLAMEN D'ASSIGNY Capucine, FLOMET Caroline, FLOTTES Yohann, FLOURIOT Anne Charlotte, FOCILLON Clara, FOKA TICHOUE Hervé, FOLTZ violaine, FONGA Sabrina, FONT Favie, FONTAINE Hélène, FONTANELLA Inès, FONTENAS Enguerran, FONTENEAU Anna, FONTI Benjamin, FORESTIER Mélanie, FORICHER Leila, FOUCAUD Pierre, FOUCHER Anne-Isabelle, FOURCADE Corine, FOURMAUX Christine, FOURNEL Sophie, FOURNET sandrine, FOURNIER CHARRIERE Elisabeth, FOURNIER Véronique, FRABOT Valentine , FRAITAG Delphine, FRAITAG Sylvie, FRANCESCHINI-MANDEL Anne, FRANCHI GERALD, FRANCHI REZGUI Patricia, FRANCOIS Eileen, FRAYSSE Nathalie, FREBAULT Emelyne, FREMOND Marie-Louise, FRERE Corinne, FREREJOUAND emmanuel, FRISCH Colette, FROHLY sylvie, FROMENTIN-JARRIER karine, FUSTIER Anne, GABAI Ilana, GADION Margaux, GAGEY Olivier, GAILLET-LAGRANGE Alyssa, GALANAUD Damien, GALANTE Florian, GALANTIER Marlène, GALIEVSKY Mathilde, GALLAS SOPHIE, GALULA gilles, GANDJBAKHCH Frederique, GANFOUDI Dhekra, GARBARG CHENON LAURENCE, GARBOWSKI Valentin, GARCON monique, GARDERE Emma, GARDERET LAURENT, GAREL Denis, GARESTIER Julia, GARIEPY JEROME, GARNIER Alicia, GAROU Caroline, GARREAU Marie, GASPARD Woody, GASSAMA Salimata, GASTON-DREYFUS Sarah, GASY Johanna, GATTEAU adrien, GATTI Celine, GAUDAIRE aurelia, GAUDART Pauline, GAUDIN Eva, GAUDRIC Marianne, GAUDRON Marion, GAUDU Charlotte, GAUDU Sophie, GAUFFENIC alan, GAUTHEY Aymeric, GAUTHIER MARIE-CHRISTINE, GAUTHIER Sarah, GAUTHIER Yoann, GAUTIER Anne Laure, GAY Françoise, GAY Vincent, GAZZOLA Morgan, GE Nathalie, GEFFRAY justine, GELLY Jennifer, GELOT antoinette, GEMIN Julien, GENDRON Pauline, GENDROT Benoît, GENIN Thomas, GENOIS Manon, GENTER LORENTE Lou, GENTIL Marion, GEOFFROY Camille, GERAL Eulalie, GERARD Erwann, GERARD Laurence, GERARD Maxime, GERARD Ysé, GERARDIN Michèle, GERENTES Mona, GERMAIN Emma, GERME Eléa, GERMOND Sophie, GHANMI Hanene, GHAOUI Amine, GHARBI DONIA, GHARIANI Elsa, GHATTASSI Zeineb, GHIGHI Maxime, GIABICANI Eloïse, GIACOPELLI Maud, GIAOUI philippe, GIARRIZZO Flore, GIBEAU Isabelle, GILLAS FABRICE, GILLE Cécile, GILLET ANNE, GILLON Flore, GINDRE Apolline, GINISTY Danielle, GIORGI Laetitia, GIRARD GUILLAUME, GIRARD-STEIN Laura, GIRODON-BOULANDET Emmanuelle, GIRONE Alexandre, GITS-MUSELLI Maud, GIUDICELLI Emma, GLICENSTEIN Frédéric, GODEFROY William, GODRON Nicolas, GOLLETY arnauld, GOMEZ Emmanuel, GONCALVES JACQUEMIN Sarah, GONTIER Arthur, GONZALEZ CANALI Gustavo, GORNET Jean-Marc, GOSSEC Laure, GOSSELIN Robin, GOSSET Daniel, GOTIN Murielle, GOTTI Nicolas, GOTZ EVELYNE, GOUDARD Philippe, GOUDJIL Asma, GOUJON Clément, GOULLEY Adrien, GOUPY sylvaine, GOURDON Capucine, GOUTAGNY Stéphane, GOUVERNAL Catherine, GOUVERT Thibault, GRABLI David, GRABLI Samuel, GRACIET ARMELLE, GRAMOND Clément, GRANDPRE Camille, GRANGE Julie, GRANGER Benjamin, GRASSIN DELYLE Aurea, GREGORIEFF Tatiana, GREMILLON Pierre-André, GRIMAUD Marie, GRINHOLTZ Alain, GROS Saga, GROTTO Sarah, GRUSON Léa, GRZESIAK Sarah, GUEGANO Madeleine, GUEHRIA Nassima, GUENIN Matthieu, GUENNEC Valérie, GUERET PASCAL, GUERIN ALMA, GUERIN Pascaline, GUERNIOU Caroline, GUESSOUM Inès, GUETTROT IMBERT Gaelle, GUEZ Nathan, GUIBOURDENCHE Jean, GUIDDIR Tamazoust, GUIGNAT Laurence, GUILLARD Mélodie, GUILLAUME-CZITROM Séverine, GUILLAUMET Gonzague, GUILLEMAIN Veronique, GUILLEMOT Eva, GUILLON Marine, GUIMOND Claire, GUIOCHON-MANTEL Anne, GUITON FANNY, GUITTARD Aurore, GUITTON Corinne, GUYENNE Anthony, GUYON Alice, GUYONVARCH Ophelie, GWENANG Francine , HABERT Marie-Odile, HADACEK Blanca, HADDOUCHE Abdallah Aylan, HADJAB Bilal, HADRI Rabab, HAGGAG Salwa, HAIM FREDERIC, HAINQUE ELODIE, HAIUN Mathieu, HALLE benjamin, HALPERIN Sophie, HAMDI Sarah, HAMMADI Manal, HAMMAMI Sanaa, HAMMOUDI Nassim, HAMOU Lauren , HAMRIOUI Idir, HANICHE Elsa, HANNETEL Oscar, HANRAS Marie, HARBA Chahem, HARTLEY Sarah, HASSAN Zeyad , HATTAB léna, HAUTEFEUILLE Eva, HAVRE Baptiste, HAZAN Fanny, HAZARD Margaux, HEC Mathilde, HECKLY Lucie, HEDDAR Abdelkader, HEIDET Laurence, HEIMANN Audrey, HENNION Christiane, HENRAS Océane, HERMAND Helene, HERMOUET Christine, HERON Delphine, HEUZE CHRISTINE, HINE Florence, HINTERLANG Mélanie, HO Bao Nghi, HO Hanh-Dan, HOARAU Wanda, HOCIANAT Enael, HOCQUEMILLER Raphael, HOLEMAN agnes, HOLZMAN Judith, HOSSARI Fabien, HOUNDONOUGBO Cécile, HOUSSET Bruno, HOZE CHLOE, HUE Sophie, HUET angele, HUGONOT LAURENCE, HUIBAN Cloé, HUSAIN Mir Dianat, HUYNH Bich-Tram, HUYNH thi Ngoc VAN, IBOS Antoine, IDBAIH Ahmed, IGOR Beleza, ILLIANO Manon, IMBAULT Marion, IMBERT Arnaud, IMBERT simonne, IN ALBON Marie, INIGUEZ Jean-luc, IPPOLITO Laure, IRAQUI Ilham, IRIGOIN-GUICHANDUT Marc, ISHAC marcel, ISNARD BAGNIS corinne, ISRAËL-BIET Dominique, ISSAURAT Pauline, IZQUIERDO Facundo, JAAFRI Siham, JACOB Julian, JACOB Paul, JACQUEMINET Sophie, JACQUESSON Laetitia, JACQUET Floriane, JACQUIER Louise, JACQUIN Paul, JACQZ-AIGRAIN Evelyne, JAEGER Clara, JAFFRES MARINE, JAMET Camille, JANICOT CAMILLE, JANKOVIC Johanna, JAULMES-BOUILLOT Helene, JAZIRI Asma, JEAN Betty, JEAN Camille, JEAN-FRANÇOIS Jordan, JEANJACQUES Hyacinthe, JEANJEAN Lilas, JEGOU Marie, JESTIN Christine, JEUNE Julie, JIMENEZ Paula, JISPHAN Isabelle, JODELET Pierre Alain, JOHN-JACOB Anne, JOLLANS Valentin, JOLLIOT Blandine, JOLLY Robin, JOLY CASSANDRE, JORUS Mélisande, JOSEPH-NOEL Maxime, JOUAN marie-claire, JOUAN Maud, JOUMELARD Ornella, JOURDAIN Arthur, JOURDAIN patrick, JOURDIN Thomas, JOUY Suzanne, JOVIAL Anthony, JUILLET Laure, JULIA Clémence, JUVIN Karine, KABLA Merryl, KADDOUR Gabriel, KADRI Youcef, KAHN Juliette, KAMIONER Phoebé, KAMIONER Thaïs, KARANOUH Rayan, KARDACHE Mourad, KARILA Chantal, KARINTHI daniel, KAS Aurelie, KASSOURI Camélia, KATAN Noa, KATONA Alix, KAZA Clément , KELLER PETROT Isabelle, KEMULA Mathilde, KERAVEL Maÿlis, KEREN Boris, KERGUEN Joséphine, KERGUENO Pauline, KERNER ALEXANDRA, KEROB Delphine, KETTANE yannis, KEV Lydie, KHA SEANG, KHALFALLAOUI Chloé, KHEDIR Sara, KHODR Adam, KHRAICHE Diala, KIMPE Jean-Pierre, KING-GILLIES Leo, KISIATA KINGUNZA ALEX, KNELLWOLF-COUSIN Anne-Laure, KOHN SCHERMAN Sylviane, KOMAJDA Michel, KOPP Pauline, KORAICHI FAIROUZ, KORB Diane, KORENBAUM clément, KORTOBA Khalil, KOSKAS Isabelle, KOSKAS Samuel, KOSSI Sandra, KOUTCHINSKY sonia, KOYUNCU Isabelle, KRASSO Marie, KREPLAK georges, KRIEF Jérôme, KRUK Hanna , L'HOMME Richard, LABAEYE solange, LABED Paul, LABIB Mouna, LABOURE Clémentine, LABRUNYE Pauline, LACAILLE Dominique, LACHHEB Mariem, LACROZE Laurence, LADHUIE Héloïse, LADJEROUD Salima, LAFOND YVES, LAGET Marine, LAGRANGE CAROLINE, LAGRAVE Constance, LAHLOU Imene, LAI Cécilia, LAISSY Jean-Pierre, LAJUS Marion, LALAUDE Olivier, LALLEMANT Pauline, LALLEMENT Tristan, LALOU Noémie, LALOUM Benjamin, LAM Nina, LAMBERT Benoit, LAMIAUX Léa, LAMOUREUX-DULAC Camille, LANDEL Léa, LANDMAN Samuel, LANGLET Alexia, LANGLOIS Clara, LANIER-ROUSSEL Marie Christine, LARBOULETTE Gaëlle, LAREDO Mikael, LAROYE Alexandre, LARRICQ Valerie, LASTENNET diane, LATOUR Mathilde, LAUNAY Pauline, LAVEDRINE Heloise, LAVENEZIANA Pierantonio, LAVERDET Stéphanie, LAVERGNE ANNE, LAVILLONNIERE Lise, LAVNER Bénédicte, LAVNER Michel, LAX Tessa, LAXENAIRE Arnault, LAYOUSS Wael, LAZARO Hélèna, LE BEYEC - LE BIHAN Johanne, LE BRAS PHILIPPE, LE BRETON FREDERIQUE, LE BRONEC Juliette, LE BRUCHEC HAYETTE, LE CAM Stéphane, LE CLEACH Laurence, LE DU Nathalie, LE FLOCH Floriane, LE FLOCH Liza, LE FLOHIC HALIMA, LE FORESTIER Nadine, LE FUR Clément, LE FUR Tomas, LE GALL Yann, LE GATT Pauline, LE GOUELLEC anne, LE GUENNEC Sylvie, LE GUYADER Anaëlle, LE HARISSON, LE HEUZEY JEAN-YVES, LE JOUBIOUX Clémence, LE Julien, LE MAIGNAN Christine, LE MEN Adèle , LE MERLUS Merlin, LE NAY Marion, LE PAJOLEC Christine, LE QUEAU Claire, LE ROUX MARIELLE, LE STRAT Laura, LE TANNEUR Marguerite , LEBAN MONIQUE, LEBAS DE LACOUR Jacques, LEBBAR Aziza, LEBELLE Anaïs, LEBLANC djamila, LEBLANC Marion, LEBON Éloïse, LEBON Lucie, LEBRUN Louis, LEBRUN Mathilde, LEBRUN-VIGNES Bénédicte, LECERF Manon, LECHAT philippe, LECLERC-MERCIER Stéphanie, LECLERCQ Julie, LECLERE Romain, LECLUSE Marion, LECOMTE Pierre, LECONTE Mathilde, LECOUSTRE LECOMTE Marie Sophie, LEDINOT Pauline, LEDOUX-PERRIGUEY David, LEFEBVRE Aline, LEFEBVRE-THOMAS Anne, LEFORT Gwenaëlle, LEFORT-LOUET Amelie , LEFRERE Bertrand, LEGENDRE Antoine, LEGENDRE Mathilde, LEGER anne, LEGRAND Anne, LEGRAND Anne, LEGRAND Carole, LEGRAND Mariane, LEGRAND PIERRE-ANTOINE, LEIGNEL sophie, LEILAZ Arnaud, LEMAISTRE Alexandra, LEMAISTRE camille, LEMAITRE DELPHINE, LEMALE julie, LEMIRE bernard, LENGLET Timothée, LENOBLE Martine, LEOSTIC Katell, LEPINE Emma, LERAT Isabelle, LERAY Sasha-Alexandra, LEROY Karen, LEROY Manon, LESAGE-MATHIEU Ninon, LESCAT Mathilde, LESCLOUPE olivier, LESIEUR Marion, LESSEUR Eva, LESTANG Pauline, LESUEUR Géraldine, LESUEUR Remy, LETANG Benjamin, LETURCQ France, LEVACHER Maryse, LEVASSEUR Joana, LEVEQUE Damien, LEVY jeannick, LEVY Richard, LHUILLIER Sophie, LIEVENS Nathalie, LIM NOYELLE Lucile, LINGLART agnes, LINGLART Léa, LO Dih, LOBUT Jean-Bruno, LOCKER Marie, LOEUR Aurélia, LOGEART Isabelle, LOGEROT Helene, LOIZEAU Maxime, LOMBARDI Fanny Françoise, LOPES Anne-Aurelie, LOPES Anne-Sophie, LORENZINI AnaÏs, LOSITO Emma, LOUBAKI TOUITOU CHLOE, LOUIS Sandra, LOURENCO Jérémy, LOURS Elodie, LOUVIER Maëlle, LUCAS GREVELLEC Christine , LUCAS Nolwenn, LUCE GARNIER Virginie, LUCHON laurent, LUCIEN Alexandra, LUCIOLLI Esmeralda, LUFEAUX Camille, LUMACA Christine, LUONG NGUYEN Minh, LUQUE Elena, LUTZ Claire, LUVET Auriane, LY Elisa, MAACHA Kahina, MACHERET Antoine, MACHI Florian, MADBOULI Nadine, MAESTRACCI Juliette, MAGNANI Alessandra, MAHAMAT ALI Sonou, MAHE MERSEDEH, MAHEUT Célia, MAHUT Bruno, MAI Thanh Van, MAIA Sofia, MAIGRET Pascal, MAILLE LAFITTE christine, MAISONOBE thierry, MAITRE Carole, MALE fanny, MALEPLATE Laetitia, MALET Antoine, MALET Jacques, MALKA David, MALKA Zaava, MALLAT Ariane, MALLET Jean Philippe, MALWE Léa, MANE Michele, MANGIN christine, MANIANI Imane, MANOLACHE CARMELLA, MARC-PERONNET Cassandre, MARCEAU Sipan, MARCHAC valentine, MARCHAL frederique, MARCHAY Nathalie, MARDALE Valentina, MARECHAL Julie, MARIE-HARDY Laura, MARIE-LUCE Elais, MARIN Alexis, MARINI Laurence, MARKOV Katia, MARLIN sandrine, MARNICHE AMEL, MARRAST Fanny, MARRE Jean-Paul, MARTEAU Anthony, MARTELLE Quentin, MARTIN AUGER, MARTIN Aurelie, MARTIN Kim, MARTIN Laura, MARTIN Nathalie, MARTIN TOUTAIN Isabelle, MARTIN-PELTIER Charlotte, MARTINEAU chloé, MARTINERIE Laetitia, MARTINEZ Christine, MARTINEZ Sarah, MARTINEZ Thomas, MARTY Tom, MARZIN Pauline, MASLE-FARQUHAR Jane, MASRI claire, MASSERA CHABOUDE Fabienne, MASSIEYE Noelle, MASSULEAU Antoine, MATHERAT Louise, MATHIEU D ARGENT Emmanuelle, MATHIEU Pauline, MATHON BERTRAND, MATTEI delphine, MATTIO Clémence, MATY ANEKEN Nancy, MAUDOUX Audrey, MAURIN Marie-Laure, MAYER Jean-Michel, MAYEUR Anne, MAZET Rose, MEBTOUCHE Sabrina, MECHALI Philippe, MEDERNACH CHANTAL, MEGHADECHA MOHAMED, MEHLER Claudia, MEILLAN Nicolas, MEILLON Laure, MEKAÏS Soufyane, MELAI nathalie, MELLET Sophie, MELOT Nicolas, MENARD Cécile, MENDY Denise, MENERET Aurélie, MERAD-WULGUE Alice, MERCADAL Lucile, MERCIER Inna, MERCIER Jean-Christophe, MERIANE Sidali, MERIGOT-BOUTIN anna, MERINE-BELARBI Linda, MERLOT Elodie, METZGER Veronique, MEUNIER domitille, MEVEL Philippe, MEYNANT Marie, MEYNIEL Perrine, MEYRONIN Ronan, MEYZER Jean-Pierre, MIAN Zain-Ul-Abdeen, MICHEL Anne-Sophie, MICHEL Maeva, MICHELETTI Léa, MICHELON Raphaël, MICHON Agnès, MICHOT caroline, MIGAULT Olivier, MIGNOT cyril, MIHOUBI fadila, MIKHAEL Gabriel, MIKOL BOURDILLAT Claude, MINEL Florian, MIQUEL Anne, MIQUEL Catherine, MIRKAMALI Amir, MKRTCHYAN Naira, MOHAMADOU Inna, MOHBAT Isabelle, MOINET Adrien, MOLAC Clémence , MOLES-ROULET Nathalie, MOLINIER Estelle, MOLLET Romane, MONCLAR Dauphine, MONNERET Sophie, MONNET Soline, MONNIER DA COSTA Aude, MONNIER-CHOLLEY Laurence, MONPIERRE Anaïs, MONTALANT Berenice, MONTANA matteo, MORACCHINI Lucile, MORAIN isabelle, MORAND Karine, MORAND Maeva, MORARDET Laetitia, MORCHAIN Antoine, MORDANT Pierre, MOREAU Tessa, MOREAUX Thierry, MOREIRA Maeva, MOREL Clémence, MOREL DE VILLIERS Alix, MORELLE Guillaume, MORETTI Léa, MORIN Clothilde, MORISSET Mathilde, MOSBAH Héléna, MOSNIER-PUDAR Helen, MOUHAMATH Hasina, MOUILLOT Damien, MOULIE Arthur, MOUNIAMAN-NARA Justine, MOUNIER Matthieu, MOUTON Elisabeth, MOUZARINE Fatima, MUCCIO Thomas, MULARSKI Agatha, MULLER Pierre, MULLONI Claire, MUNCK Anne, MUNCK Jean-Nicolas, MURAT Quitterie, MURE Agnes, MUSSAT Philippe, MUSSO Elsa, MUTTERER Inès, MYRTIL Christelle, NACER Salma, NAFOUTI Neissene, NAIIM Isabelle, NAIT-IGHIL LELLA, NAJEAN Marie, NAKACHE Emmanuel, NAPPEZ Muriel, NASONE Justine, NASSEF GUERARD Fatiha, NASSIRI Kourosh, NATAF joelle, NATHAN Nadia, NATIVEL Elisa, NAVARRE Alix, NAVARRO Soledad, NAVETIER Louis, NDIAYE GUEYE DIARIETOU, NECTOUX Juliette, NEGRE Axelle , NERVO Marine, NETCHINE Irene, NETO SOP OCÉANE, NEURAZ Annick, NEVES Coralie, NGUTUKA Mélanie, NGUYEN Florence, NGUYEN François, NGUYEN KHAC Florence, NGUYEN Lan-Anh, NGUYEN Quentin, NGUYEN SON Caroline, NGUYEN SYLVIE, NGUYEN THI Hoai Huong, NGUYEN Thomas, NGUYEN THU NHI, NGUYEN-KHOA Jean-Luc, NICOT Florence, NICOULES Laura, NIEBEL Yves, NIEZ Natacha, NIOPEL Romane, NISKY Arnaud, NISSAN Caroline, NOBILET Loup, NOEL Marion, NOEL Olivia, NOEL PETROFF Nathalie, NOKAYA Stéphane, NOROL Francoise, NOROY Dominique, NOUAILHAC Emeric, NOURI Mohamed Refaat, NOURI Shama, NOWAK Alexia, NOYELLE Pierre-augustin, OBADIA Anaelle, OBTEL NICOLAS, OGHEARD Emma, OHAYON Salomé, OLDERS Eléonore Marie, OLUGBEJE Helen, ORFILA Guillaume, ORLIK Benjamin, ORMEN Alice, ORMIERES Clothilde, ORO Hubert, ORO Saskia, ORTOLAN Dominique, OSIKA Eric, OUAHBI Abdeljalal, OUAHIDI Karine, OUELD Eva, OUESLATI Ilann, OUTENAH Cécilia, OUZEGDOUH MAYA, PAITIER Caroline, PAME Patricia, PAMUK Gizem, PANAIOLI Elena, PAOLANTONI Pholippe, PAPILLON Laura, PARENTELLI Anne Sophie, PARK Sun-Young, PARMANTIER Léa, PARMENTIER SABINE, PARMENTIER THIERRY, PARODI Marine, PARREIRA Mélanie, PASCAL Ludwine, PASCAUD Maxime, PASSET Marie, PASTEL Aïda, PATALAKH Polina, PATRAT Catherine, PAULIAT-DESBORDES Sylvie, PAULIN Hervé, PAVARD-BRUN Alexandra, PAYAN Samantha, PECHINE Manon, PEENE Héléna, PEIFFER Claudine, PENAUD Dominique, PENGUE KOYI Anais, PENSEDENT Adeline, PENSO-ASSATHIANY Dominique, PENTEL Jonathan, PEREIRA Eva, PEREIRA Johanna, PERETTI Quentin, PEREZ EPOUSE WEILL Martine, PEREZ Valérie, PERGELINE Jeanne, PERNET Yuna, PERRAULT DE JOTEMPS Arnaud, PERRIAU Océane, PERROY Marie-Dominique, PERRY Ariane, PERTHUIS Valentin, PERVES KECHEMAIR CATHERINE, PESQUE Louise, PETEYTAS Anouck, PETIT DAMICO Inès, PETIT Lola, PETIT Sylvain, PETITFILS Arnaud, PETKOVA Elena, PETRETO Tiffany, PETRISSANS Mayia, PETROU Elodie, PEYRE Matthieu, PEYRO SAINT PAUL Helene, PHAM QUANG long, PHAM-HI Minh-Ha, PHILIPPE Aude, PHLEK Narada, PICHIT Phintip, PICHON Tiphaine, PICOT Geneviève, PIDET Olivier, PIERARD Thibault, PIERGA Alexandre , PIERREL Blueberry, PIETTE FRANCOIS, PIETTRE Anaïs, PIEVIC Clémence, PILCH Manon, PINANA Margaux, PINCHEMEL Marion, PINTON Anne, PIOLINE MAUD, PIOT Béatrice, PIRABAKARAN vithura, PIROLLO Laura, PIRON Marion, PITON Julie, PIVETEAU Sabine, PIZZUTI Melissa, PLACOTARIS Gabriel, PLANES CHARPENTIER Veronique, PLANTARD SOPHIE, PLONQUET Anne, PLOQUIN Laurence, PLUMEY-CARBONNIER Veronique, PLUVINAGE AMELIE, POCATE Khaled, POCQUET Chloé , PODWOJEWSKI Laura, POLINE Julie, POLIVKA Laura, PONG Kolianie, PONTICQ Francoise, PORTERO Raphaël, POTHIN PATRICIA, POTOT Aurélien, POULAIN Christophe, POULAIN-FERARIOS Camille, POULARD Sylvie , POULIQUEN Françoise, POURCEL Isabelle, POURCELOT anne gaelle, POURMOMEN ARABI Kyan, POURQUE Bénédicte, POURRIAT JEAN LOUIS, PREBET Coralie, PRETA Laure-Hélène, PREUD'HOMME Bastien, PREVOST Blandine, PRIEUR Elodie, PRIGENT Jade, PROISL Oliver, PROUZEAU-AUGUSTIN Sylvie, PUEYO (EP VELHO) Maria, PUJADE-LAURAINE Eric, PULL Lauren, QATRAMIZ Ahmad, QUACH Marie, QUENET Gwenola, QUESTROY Magalie, QUEVAL Antoine, QUINCAMPOIX Caroline, QUINSON Emilie, RACY Camille, RADZIK Anna, RAFENOMBOLATIANA Camille, RAGANI Clara, RAHERIARIVELO Mendrika, RAHMOUNI Justine, RAMBOANASOLO Joelle , RANARIJHON Eymeric, RANSON Marine, RAPHAEL Ny, RATHAMOHAN Grecinta, RAUDE GARRET Catherine, RAYMOND AUSSERT Frederique, REBIERE Lucille, RECANATI Franck, REGNIER Elodie, REGNIER Océane, REIGNIER victor, RELANCIO TEIXEIRA FILES Mathilde, REMY Suzanne, RENARD Cindy, RENARD PENNA Raphaele, RENE-CORAIL Diane, RETAILLEAU Jeanne, RETBI Isabelle, REVAH Alain, REY Pierre-Antoine, REY-COQUAIS CECILE, REY-WILISCH Michèle, RIANT Marie, RIAUDET Enimie, RICHARD Pascale, RICHARD Sandrine, RICHAUD Albane, RICHAUD MARC, RICHET Anne-isabelle, RIDET Lydie, RIGAULT Eve, RIGOURD virginie, RIMBAULT frederic, RIO marlene, RIOUAL Bénédicte, RISOLO marc, RITTER Charlotte, RIVIERE Emeline, ROBELIN Lucas, ROBERT DE RANCHER Marie-Aude, ROBERT Valerie, ROBERTSON Alix, ROBIN Alexandre, ROBIN Nicolas, ROBLOT Victoire, ROCHER Nicolas, RODA Nathalina, RODRIGUES Mélanie, ROFFI Fabio, ROGER Anissa, ROLLAND Benedicte, ROLLAND Zoé, ROMANA Serge, RONEZ Emily, ROQUETTE ALIX, ROSELIER Noëllie, ROSENCHER Lise, ROSPABE Matthieu, ROTARU Irina, ROTENBERG Beatrice, ROTSEN Jacques, ROUAULT Marion, ROUFATI Imène, ROUFE Elisa, ROUMANI Moad, ROUQUETTE Cyrielle, ROUSSARD Philippe, ROUSSEAU Aurélie, ROUSSEL Camille, ROUSSEL Perrine, ROUSTANG Isabelle, ROUX Carole, ROUX HURTAUD Marie-Françoise, ROUX Sabine, ROUXEL AGNES, ROVANI Sibylle, ROYER Catherine, ROZ Alexandra, ROZE JULIE, ROZENBERG sylvie, RUAUD Lyse, RUFAT Pierre, RULLAN BORRAS Margaux, RUPRICH-ROBERT Gwenaël, SABATINI Pauline, SAHRI Lina, SAIDI Yanis, SAÏSSE Rachel, SAJOUX Hippolyte, SALEM clara, SALFRANT Maxime, SALIBA Marie-Charlotte, SALMON Remy, SAM Lynda, SAMAMA Mickaël, SAMAMA pascale, SAMBA Célia, SAMIRI Maïssane, SAMOUN Shirel, SAMY Laetitia, SAN SEBASTIAN Axel, SANDRÉ Christine, SANDRE Pauline, SAOUNERA Diana, SAPORTA SYLVIE, SARACINO Dario, SARFATI Sarah, SARINA-NEWMAN Aktoty, SASSINOT Louis, SAUVAN marine, SAUVE MARTIN Hélène, SAUVEPLANE Manon, SAVANNE loic, SAVEANT Pénélope, SAVY Loreena, SAWKA CHRISTELLE, SAYAG Anaelle, SAYED Mohamed, SCEMAMA Guy, SCETBON Gilles, SCHAUER Florence, SCHIFF Manuel, SCHINDLER Sophie, SCHINKEL Somnah-Nelly, SCHLEICHER Lucia, SCHLEMMER Benoit, SCHLUMBERGER Marion , SCHMIT Quentin, SCHMITT Emilie, SCHROEDER Justine, SCHUMACHER Cyril, SCIALOM Leslie, SCOLARY CLAIRE, SEBAA Karim, SEBAG Ethel, SEBAHOUN Stephane, SEE Hélène, SEHNAL Pauline, SEILLER Astrid, SELLEM Corinne, SELOSSE Christelle, SEMENNIKOVA Katherine, SENELLART Clara, SENOUSSI Laurence, SERENI carole, SERENI daniel, SEROR Adrien, SERRURIER Mélodie, SERVOLIN Marianne, SERVY Amandine, SETTI Benjamin, SEVEYRAT Raphaël, SEZIKEYE MAGUELONE, SFEIR Alexandre, SHARIFZADEHGAN Shayann, SIBERT annie, SIDIBE Fatoumata, SIFFLET Christophe, SIMOES Catarina, SIMON Anne Laure, SIMONIN Mathieu, SIMONNEAU Gerald, SIOUTI Clara , SIROL Marc, SIRVEN-VILLAROS lila, SISSAOUI SAMIRA, SLIMANI Hind, SOBOL Edgar, SOHEILI fabrice, SONSOIS Cheyenne, SOUAID Carl, SOUBEYRAN Clément, SOUCHET laetitia, SOUIED Hanna, SOUMAH Mariam, SOYER Aline, SOYER Yves, STANCIU Rica, STANKOFF Bruno, STEIN sophie, STEVE Marc, STEVENS Jerome, STHENEUR Chantal, STIELTJES Natalie, STIVALET Olivier, STOECKEL Jeremy, SUAREZ ROOS Lena Gabriella, SUISSA Yona, SULUKDJIAN Arek, SULZER Philippe, SUPRIN Céline , SUY Sorya, SZCZEPANSKA GONZALEZ Margot, TABATH Martin, TABO David, TABTI DUPRE KARIMA, TACHE Amokrane, TACK Veronique, TAHAR-CHAOUCHE Louiza, TAIEB ANTHONY, TALABARD Marie Pauline, TALLON Pauline, TAMBOURINDEGUY Marion, TAMISIER Clara, TAN marina, TARDIT Thomas, TASSIN Mikaël, TAT Ionut, TAVARES RAMOS Laurence, TAWFIK Muriel, TEILHET Camille, TEIXEIRA Antonio, TENENBAUM Annabelle, TENENBAUM florence, TENENBAUM nora, TENGHER BARNA IULIA, TERMEAU STEPHANIE, TETTEKPOE Margaux, TEXIER Alexandre, TEYCHENE anne marie, THAI Stella, THEBAULT Robert, THEROND Justine, THEROND Romane , THERY Lisa, THEVARAJAH Mary, THEVENIN Juliette, THEVENON Joannice, THIBIERGE ELISABETH, THIMON Coraline, THIONGANE Rokiyatou, THIRION Clément, THOMAS Daniel, THOMAS Jean-Luc, THOMAS Laura, THOMAS TEINTURIER Cécile, THOUVENIN Guillaume, TIMERA Hawa, TIMSIT françoise Julie, TIMSIT jose, TIXIER Camille, TO Ella, TOLEDANO Cecile, TOLEDANO Lisa, TOMBERLI Françoise, TOMCZYK Laetitia, TON Virginie, TONDELIER Clémence, TOSTI Grégory, TOUBIANA Chloe, TOUITOU Laurence, TOUIZER-BENAROCHE Emmanuelle, TOULEMONDE Béatrix, TOULMOUTINE Ines, TOURIGNY Camille, TOURNIER Apolline, TOVAR Celia, TOVARNITSKA Oksana, TRAN Béatrice, TRAN Marie France, TRAN VAN NHIEU Jeanne, TRAORE CYRIL, TRAORE FATOU-NANA, TRISTANI Baptiste, TROADEC Benoit, TROCELLO Hugo, TROCHON Manon, TROLLIET Marie, TROUVIN Jean-Hugues, TUBERY Amandine, TUKA Debora, TURE Fatoumata, TYSEBAERT Julie, ULIAN Capucine, UZAN monique, VADROT Caroline, VAHANIAN alec, VAISLIC Muriel, VALDES Lydia, VALENCIENNES Julie, VALERY charles, VALLA Dominique, VALOT Anne, VAN DER STRAATEN Axelle, VANDER-HEYM Chloe, VAPPEREAU Margot, VASNIER Christelle, VASSEUR Alice, VEBER FLORENCE, VEDRENNE Geraldine, VEDRINE Claire-Marie, VELLAR Marlene, VENDITTO marcia, VENENCIE Pierre-Yves, VERCELLINO LAETITIA, VERGEZ Mylene, VERGIER romain, VERNILLET Romain, VERVAEKE Nathalie , VERY Géraldine, VESIN Justine, VEZIRIS nicolas, VIALA Karine, VIARD Camille, VIAUX Sylvie, VICTOIRE Lindsay, VIDAL Estelle, VIDAUD Dominiaue, VIDIL Anne, VIEUBLE Laura, VIGNAUX Eloi, VIGNERON jeannette, VIGOUROUX Corinne, VILLAIN Chloé, VILLART Agnès, VINCENOT anne, VINCENT denis, VINCIGUERRA CORINNE, VINOLAS Hélène, VINSONNEAU Laura, VIREVIALLE Clemence, VIRLOUVET Audrey, VISEU GOMES Cynthia, VISSAC Fanny, VITTECOQ AURELIE, VITTECOQ Daniel, VIVIER Lucie, VO Hong, VO TRAN Ai Anh, VOGEL-LEDAIN Isabele, VOISIN Nicolas, VOLAND Cécile, VONNER Thomas, VOZY aurore, VRANGOS Juliette, VROUST Tracy, VU THAN Amélie, VUILLEZ Arnaud, VUONG Laurence, WAKIM Marc, WAKIM Michel, WALDBURGER Valentine, WALSER Sébastien, WANG Sébastien, WARNECK Léa, WEIL Victor, WEILL dan, WEILL-ENGERER Sébastien, WILLEMS lise, WILLMES Marion, WISE Scarlett, WOLTER-DESFOSSES Michèle, WORBE Yulia, WROBEL Jacques, WULFMAN Claudine, WYBIER Nathan, XANDRY MATTHIAS, YAACOUB Quentin, YONNEAU Jules, YSEBAERT Mélissa, YUNG Emilie, ZAGORI Ivan, ZALUSKI Daniel, ZAMBON Bernadette, ZARKA LORAINE, ZAYOUD Ayla, ZAYOUD Ninar, ZEE Nathalie, ZELL-VAILLANT Benjamin, ZENKHRI Ferielle, ZENNARO Maria-Christina, ZEROUAL Lamia, ZHANG HU Ying, ZHAO Céline, ZINTCHEM Roger, ZOUQI Nicolas, ZUGOLARO Maxime, ZYLBERSTEJN Ines,

*Including physicians:* the ABBES Yamina, ABEILLE Vincent, ABERRANE Achour, ABICHOU-JELOUALI Amel, ABITTAN-SMADJA Jennifer, ABLARD Jacques, ABTAHI Mahdi, ACHE Sandrine, ACHKAR Wissam, ADELINE-DUFLOT Florence, ADER Penelope, ADRAR Ryad, AGAR-BOULAABI Amel, AHMADI Sadaf, AISSOU Linda, AIT MAAMAR Nadia, AKHERRAZ Malika, ALARD Isabelle, ALBERT Francis, ALDEGUER Guillaume, ALEXIOU Anna, ALIANE Nawelle, ALIMI MARDOUKH Eleonore, ALIZON Jean-Pierre, ALLAIN Olivier, ALLALI Yann, ALLOING Lila, ALLOUCHE Catherine, ALLOUL-TOLEDANO Irene, ALVARADO Rafael, AMABILE Marie-Helene, AMANOU Laurent, AMAR Paul, AMARGER Frederic, AMAT Valerie, AMAZZOUGH Karima, AMBIEL Jean-Philippe, AMMELOOT Jean-Marc, AMOKRANE Sonya, AMOR CHELIHI Lahoueri, AMSELLI Axel, ANDEL-AMIEL Audrey, ANDRE Christophe, ANDRE Sandrine, ANDRE-ARLET Helene, ANDRO Claire-Marine, ANTOGNARELLI-DUBREUIL Lena, ANZIANI-VENTE Gabrielle, AOUSTIN Nadine, ARBAOUI Samia, ARBOUCH Cecile, ARDITTI Laurent, ARETZ-TAIEB Nicole, ARISTE-ZELISE Celine, ARIZTEGUI Albin, ARMAND Jean-Paul, ARNAUD Frederic, ARNAUD Ludovic, ARNOLIN GUANNEL Marie-Christine, AROUMOUGAME Vani, ARSLAN Erkan, ARSLAN Maria, ARTIS Coralie, ASKIN Dilek, ASSIMADI Yao Ameko, ASSRIH Meriem, ASTRUC Alexis, ATEMKENG NANFACK Georgette, ATHIAS Thierry, ATKINS Karine, ATLAN-COTTIN Audrey, ATOUT-BEN MOUHA Saida, ATTAF Sihame, ATTAL-BEHAR Julie, ATTALAH Mona, ATTALI Claude, ATTIA Laurent, ATTIAS Alexandre, AUBERT Jean Pierre, AUBERT Jean-Maurice, AUBERT-LE GRIMELLEC Delphine, AUBIN-AUGER Isabelle, AUBRY Bernard, AUDEBOURG Pascale, AUDRAN Ghislaine, AUTRE Medecin, AVANTURIER Judith, AVENEL Gerard, AVENIN Guillaume, AVOGADRI-BOYER Corinne, AWADI Samir, AYDIN Jean-Marc, AYME Nadine, AYOUN Gilles, AZERAD David, AZOULAY Eric, AZOULAY TACHON Maud, AZY Suleiman, AZZOLA Valerie, BABULE Veronique, BACCHETTA Vittorio, BACH Lorene, BACHLER Eric, BACLE Francoise, BACQUER Alain, BADAOUI Malika, BADIANE Ndoumbe, BAES Mathilde, BAH Hassimiou, BAH-ASSOUMANI Salamata, BAILLON-BOTTEAU Gwladys, BAKER Catherine, BALIGOUT Aurelie, BALYELI Filiz, BAMBERGER Marion, BANOUN Eric, BARANES Charles, BARBAROUX Sebastien, BARBERGER-SEBAOUN Florence, BARBERY Laurence, BARILLE Elodie, BARISSAT-LEGRAND Carine, BARON-BERTRAND Stephanie, BARRAT Eric, BARRE Jerome, BARRE Paul, BARTHELEMY Cecile, BARTHELEMY Pierre Ludovic, BARTHEZ Philippe, BARTOLETTI-ARTIGES Claire, BASEYA Amelie, BASSEPORTE Julien, BATAILLE Catherine, BATARD Christophe, BAUDRIER Thierry, BAUTAIN-DURAND Sonia, BAZIN Lea, BEAUCHAMPS Bruno, BEAUNIER Philippe, BECAM Fatoumata, BECCHIO Tatiana, BECEL Emmanuelle, BECHET Frederique, BEDAT-DURAND Colette, BEDEL-CHAUVAUD Julie, BELFORD Martine, BELHOUARI Katia, BELKACEM Fatiha, BELKAID EL MOBARIK Siham, BELLAICHE Sabine, BELLEUDY Michel, BELLEUDY-SOMBSTAY Claire, BELLEUVRE Florence, BELLIER-CAUSSIN Nathalie, BELLON Amelie, BELLUT Pascale, BELOTTE Michelle, BELOUCHI Mustafa, BELTRAMO Claire, BEN ABDALLAH Farid, BEN REHOUMA Bassem, BEN SAID Jean Louis, BEN SALAH Loubna, BEN SASSI Brahim, BENACHENHOU Faliha-Ghita, BENAIS Jean-Pierre, BENAMOUT Georges, BENAMOUZIG-GOLDRAJCH Eliane, BENARBIA Yassine, BENASSAIA Eric, BENATAR Yoann, BENBRAHIM Zilassen, BENCHIMOL Bernard, BENHAMRON DIT ZBILI Beatrice, BENICHOU Luc, BENIL Nathalie, BENITAH Elsa, BENOIST Luc, BENOIT Jean Michel, BENOUDA Leila, BENOVICI Didier, BENQUE Marie, BENSALEM Nabil, BENTALEB Abdelghani, BENVENUTI Christophe, BENZAKEN-TERNON Suzy, BEQUET Laurence, BERCOVICI-TOUSSAIN Sophie, BERIOT Marie-Rosaire, BERKACHE-KHOUAS Kenza, BERKAI Rania, BERLINER-KAROUBY Isabelle, BERNARD Corinne, BERNARD Francoise, BERNSTEIN Marion, BEROU Pierre, BERRADJ Salim, BERREBI Paul, BERRIER-FASANINO Juliette, BERTEAUX Sebastien, BERTHELOT Dominique, BERTHET-BONDET Philippe, BERTIN Laurent, BERTIN Yves, BERTOLIATTI Sylvie, BERTRAN Segolene, BERTRAND Francis, BERTRAND Pierre, BERTRAND-DESVAGES Genevieve, BERTRANDON Laurence, BESSE Cecile, BESSIS Alain, BIBAS Aurore, BIBAS Jordan, BIDERMANN Marion, BIEBER-HATRY Marie-Laure, BIEN Borivan, BIGNANI Olivier, BILLAUD Julie, BILLAUD Max, BILLOT-HADANA Stephanie, BILLOTTE Heloise, BILLY Isabelle, BILTZ Pascal, BINET Herve, BINET Marie, BINET Priscilla, BIRENE Richard, BIRON Audrey, BISI Laura, BISIO Martina, BISSONNIER Severine, BITAR Dounia, BITTON-HORYN Carlynn, BITTON-REZLAN Danielle, BLANC Cecile, BLANC Jean-Baptiste, BLANC Timothee, BLANCHARD Thierry, BLATANIS-BANCILLON Brigitte, BLAVAT Anne-Marie, BLED Marine, BLIN Dominique, BLIN-DE FILIPPIS Marie, BLIVET Sandra, BLOCH Deborah, BLOIT Dominique, BLOND Pierre-Andre, BLONDEEL Eleonore, BLOT-HAVETTE Sylvie, BOFFA Claire, BOILEVE Victor, BOISARD Camille, BOISSERAND Luc, BOISSON Sylvie, BOISVIEUX Thibaut, BOMMENEL Lucie, BONNAUD-PORAS Helene, BONNAVE Nathalie, BONNEMAISON Patrick, BONNOTTE Gaelle, BONTEMPS Florence, BONVARLET Francoise, BORDEREAU Marine, BORDES Charlotte, BORLETEAU DARDENNE Julie, BORRE Catherine, BORREL-VINCENT Isabelle, BORUCHOT Bertrand, BORUCHOT Boris, BOSSE Catherine, BOTHNER Laurent, BOUCHOT Christian, BOUCHOUCHA Philippe, BOUIGHAMEDANE-GALLAIS Fadma, BOUILLARD Dominique, BOULENGER Nathalie, BOULET Isabelle, BOUNGOU Marie-France, BOUQUIAUX Barbara, BOURAZI Abdelrani, BOURBON Emilie, BOURBOTTE Laurent, BOURGEOIS Claire, BOURGEOIS Francoise, BOURGES GUERRY Emilie, BOURGUIGNON Nelly, BOURGUIGNON-VARTANIAN Veronique, BOURRION Bastien, BOURROUSSE Anne-Sophie, BOUSQUET Cecile, BOUSSARD-LEFEBVRE Anne, BOUTILLON Antoine, BOUVET Sabine, BOUVIER-LECLERCQ Catherine, BOUYSSET Marine, BOYER Laurent, BRAILLARD Christian, BRAKA-HASSAN Deborah, BRAMI Jean, BRAMI Yoann, BRAY Sophie, BRES Benoit, BRET Laure, BRIDET-TARI Christine, BRIKI Rachid, BRINZA Mihaela, BRISSE Florence, BRISSEAU Jean-Luc, BRIZOU Patrick, BROCAIL Pierre-Emmanuel, BRODBECK Aurelien, BRODBECK Marina, BROQUET Philippe, BRUCKER Aude, BRUNA Sylvie, BRUNET-SABAT Laure, BRUNON Severine, BRY Marie-Laure, BUAILLON Antoine-Philippe, BUCCHIA Sophie, BUDOWSKI Max, BUGE Mickael, BURCKLE Noel, BUSSCHAERT Melanie, BUTEL Caroline, CABANNES Auriane, CABRERO-BERTEL Ines, CABRITA Sylvie, CADO Didier, CAHEN-REGNAULT Isabelle, CAHOREAU Roger, CAJEE Nawra, CALFOND-TACHON Claire, CALIEZ Philippe, CAMHAJI Olivier, CAMPANA Anita, CAMPOS Stephanie, CAMPOS-RICHARD Anna-Maria, CAMUS Heloise, CANARD Philippe, CAPITANT Patrice, CAPOCCI Gerard, CAPPANERA Remi, CAPUS Sophie, CARPENTIER Stella, CARRE Maud, CARRE-CRETOIS Sophie, CARREAU Maylis, CARRERA Carole, CASTANEDO Gerald, CASTEX-LEFEBVRE Mireille, CASTRO Corinne, CATALA Mathilde, CATTAN Fleurette, CATU Annie, CAUET Celine, CAUMONT Alain, CAVAILLE-COLL Eric, CAVALERIE Jean-Lin, CAVILLON-AGNIEL Beatrice, CAZARD Sylvain, CAZENAVE-LACROUTS Eva, CAZES Frederique, CAZIVASSILIO Denise, CERBAH Djazira, CERTES-CILLI Patricia, CERVERA Valentine, CESARI Marie-Christine, CESSOT Gilles, CHAHINIAN Michel, CHAMBRIER Solange, CHAMPENOIS Antoine, CHAN FAN Michael, CHARENT Isabelle, CHARLES Camille, CHARLES Lucie, CHARLIER Christine, CHARLON Yannis, CHARRIER Camille, CHARRIN Emmanuelle, CHASSAGNON Marianne, CHASSANG Martin, CHAUMONT Patrick, CHAUVEAU Valerie, CHAUVIN Jean-Philippe, CHAVAGNAT Julie, CHAVANNES Barbara, CHAWKI Hamza, CHAZAUD Carole, CHAZOUILLERES Anne-Francoise, CHELLAL Karim, CHEMLA Catherine, CHEMLA Emilie, CHEN-ZEE Estelle, CHENG Seav Leng, CHENIOUR-BIBI Soumaya, CHERET Hugues-Emmanuel, CHERMETTE Solange, CHERRIER Florence, CHETRIT Michael, CHEVALIER Helene, CHEVALIER Magali, CHEVALIER Pierre, CHEVALLIER Frederic, CHEVILLARD Marie, CHEVREUIL Romain, CHHIM Solara, CHIARENZA Arnaud, CHICHA Patrick, CHIVILO Isabelle, CHORT Carine, CHOUAIB-MORJANE Asma, CHOUGNET Jeanne, CHOUQUET Mathilde, CHRIQUI David, CHRISTIDIS Anna, CHURLAUD Jean-Michel, CISSE Johana, CLAIR Marie-Philippine, CLAUX Jean, CLAVERIE Claude, CLAVIE Delphine, CLERC RENAUD Alain, CLOUET Daniel, CLUET Sandrine, COCHARD Sylvie, COCHERY Guillaume, COCO Mathie, COCOUVI Solene, COEYTAUX Frederic, COFFINET Andre, COGNE Sebastien, COHEN Johan, COHEN Jonathan, COHEN Pierre, COHEN-WALTER Ilana, COLAS-DOLIGEZ Nathalie, COLLIGNON-PORTES Rachel, COLLIN Lauriane, COLOMBIES Corinne, COLONNA-AMARA Fatima, COMMUNEAU-BERTIN Brigitte, COMPTE Myriam, COMTE Laurent, CONDE Catia, CONESA Jean-Marie, CONNAULT Thierry, CONNOIS Delphine, CONTI-BOUTILLER Christine, COPPARD Marie-Pierre, COPPEL Marie-Helene, CORBI Herve, CORCHIA Laurene, CORDEL Jean-Pierre, CORDONNIER Francois, CORNUAULT Nadine, CORVAL PETITJEAN Anne-Marie, COSSON Stephane, COTAYA Joseph, COTTEN-CAVALIERI Marguerite, COUADAU Thomas, COUDERC Emilie, COUDERC Francois, COUFFON Benedicte, COULANGE-BODILIS Helene, COULON Nathalie, COUMAR Ouma, COUQUE Alice, COURVOISIER Natacha, COUSTY-FRUGIER Elisabeth, COZIAN Nadine, CREUTZER Estelle, CROCHETON Nicolas, CROCI Elodie, CROISILLE Stephane, CROIZIER-CHANIOT Ozanne, CROZAT Cecile, CROZIER Cecile, CUENCA Marine, CUESTA Juan, CUSSAC Fanny, D'AVRAY Arnaud, D'HONDT Carole, D'OVIDIO PANIS Nadia, DA CUNHA-LOUBIES Marie-Jose, DACQUIN Emmanuelle, DADOUN Sabrina, DAGHOUANE Ali, DAGOUAT Jean-Baptiste, DAGUILLON-TORRE Charlotte, DAHAN Emmanuel, DAHANE Nasr-Eddine, DAIX Laurence, DAMBOR Anne, DANG Christophe, DANG DO TRINH Thi, DANG-VU Kinh-Quoc, DANIN-MICHEL Blandine, DARDEL Nicole, DARDENNE Nathalie, DARIER Franck, DARMON Alexandre, DARMON-TRINQUET Michele, DAUMAS Matthieu, DAVEAU Pascale, DAVID Jerome, DAVIDEAU Serge, DAYRAT Patricia, DE ANDRADE Julie, DE BARY Louise, DE BECO Antoine, DE CARNE-CHANCELIER Catherine, DE CHEFDEBIEN Marine, DE COURTEIX Charlotte, DE FERRIERES Armelle, DE KERDANIEL-ARICHE Irene, DE LA DEBUTRIE Isabelle, DE LA FRESNAYE Monique, DE LA ROCHEBROCHARD Armelle, DE LAUZUN Isabelle, DE LICHANA Segolene, DE OLIVEIRA Maria, DE PONTHAUD Lucile, DE QUINCEROT Anne Charlotte, DE ROQUEFEUIL Florence, DE SA MOREIRA Edith, DE SAINTE LORETTE Eric, DE SCHRYNMAKERS Fabien, DE SILVA Nimalie, DE ZELICOURT Tiphaine, DEBAIN Chloe, DEBANDT David, DEBAS Manon, DECAMPS-LE CHEVOIR Joelle, DECK Laura, DECLEIRE-GUILLAUME Anne-Sophie, DECOUZON Herve, DEFAIX Christophe, DEFER Dominique, DEFINS Laurence, DEGEORGES Martine, DEHENT Edwige, DEIS DE FABIANI Stephanie, DEJAUNE Pascaline, DEJEAN Olivier, DEL BUONO Beatrice, DELAET Caroline, DELAETER-FICHEZ Anne, DELANDRE Arnaud, DELANOE Clelia, DELATOUCHE Saholy, DELAVAL-MOLKO Agnes, DELAVAUD Sebastien, DELAYAC Laetitia, DELEBECQUE Albert, DELL ISOLA Severine, DELMAS-GUILLOIS Benedicte, DELOFFRE Josyane, DELONG Christophe, DELORME Danielle, DELUZE Alain, DEMAY Jonathan, DEMESY Elisabeth, DEMONFAUCON Muriel, DENAMBRIDE Alice, DENAT Olivier, DENIS-DIETLIN Audrey, DENNEBOUY Christophe, DENRY Amelie, DEPONT Catherine, DERBANNE Virginie, DERHY Yves, DERRAR Naima, DERVAUX Virginie, DESBOIS Lorene, DESBONNET Francois, DESCRIMES Nathalie, DESMOULINS Frederic, DESTELLE Jean-Marie, DESTOT-VONG Kim Da, DETRE-FELIX Ophelie, DEULEU-ZIME Flore, DEVALLIERE Franck, DEVARS DU MAYNE Marie, DEVATINE Bernard, DEVILLERS Louise, DEVINANTE Aurelie, DEVORT Thierry, DEYRIS Laurent, DHEZ Catherine, DHOURY Brigitte, DI GIUSEPPE Anna Maria, DI MASCIO Patricia, DIA Papa, DIANOUX Jean Louis, DIBO-COHEN Larry, DIDON Marc, DIEMUNSCH William, DIJOLS Aurelie, DIONIS DU SEJOUR Pierre, DIOP Maeva, DIOP Yacine, DJABELLA Sonia, DJAVIDI Azadeh, DO DANG Delphine, DO Essivi, DOBROWOLSKI Benoit, DODILLE Laurence, DOMERGUE-THAN TRONG Elisabeth, DOMINJON Laure, DONIKYAN Berdje, DONNE Eric, DORANGE Aurore, DORCHE Bernard, DORE Beatrice, DORISON Francois, DOUCOURE Anne, DOUKHAN Ilana, DOULAKI Mohammed, DOUTHEAU Jean-Michel, DOUZILLE Marie-Beatrice, DRAY Sigal, DRIESCH Marie-Jeanne, DROULERS Angele, DRUON Fabien, DU BOUETIEZ DE KERORGUEN Alain, DU BREUIL HELION DE LA GUERONNIERE Florence, DU FAYET DE LA TOUR Charlotte, DUBEAUX Pierrick, DUBOIS-ROUSSEL Renan, DUBOURG Mathieu, DUBREUIL Nicolas, DUBUS Jean-Marc, DUC Philippe, DUCHOLET Anne-Catherine, DUCLOUX Francoise, DUCOURET Hortense, DUDA Monique, DUFAIX Aline, DUFAY Maria, DUFAYS Delphine, DUFOUR Carinne, DUGARDIN Bertrand, DUHOUX CASQUAT Berengere, DUMAS Olivier, DUMONT Gregoire, DUMONTEIT Amandine, DUNAND Aude, DUPIE Isabelle, DUPIN Elise, DUPLAY Camille, DUPOISOT Olivier, DUPOUX Arnaud, DUQUAY Serge, DUQUENNE Isabelle, DURAND Adrien, DURAND Claire, DURAND Francoise, DURAND Thierry, DURAND-LLEDO Marie-Ange, DURAND-SABATE Christine, DUREL Olivier, DURIEZ-MISE Gaele, DURRIEU DE MADRON Anne Laure, DURRMEYER Olivier, DUSAUSSOY Helene, DUSSAUGE Jean, DUSSAUZE Yves, DUTHEIL Cyrielle, DUVAL Helena, DUVILLIER Jerome, DUZERT Thomas, A Supprimer, AARON Emmanuelle, ABAJJANE-SARI Zakia, ABBAS Leila, ABBOU Aziz, ABBOUD Imad, ABDEL AL Anas, ABDELJAOUAD Mohamed, ABDELLAOUI Faouzi, ABECASSIS Morgane, ABEDJOUNA Amir, ABEILLE Fanny, ABGRAL Maelig, ABGRALL Sophie, ABISROR Noemie, ABITBOL Vered, ABOUAB-KARSANTI Anastassja, ACKERMANN Felix, ADAVANE-SCHEUBLE Saroumadi, ADDOU Beghdad, ADEDJOUMA Amir, ADNET Fr√©d√©ric, AFCHAIN Pauline, AFDJEI Ali, AFRIAT Jonathan, AGATHE NERINE Nais, AGRANAT Pascal, AGUADISCH Elise Elene, AGUILAR Claire, AGUIR-MOULAY Kheira, AGUT Sophie, AILIOAIE Oana, AIM-EUSEBI Am√©lie, AIME Adeline, AIT BOUDAOUD Amel, AIT-BACHIR Leila, AKEKE Marion, AKKOUCHE Farid, AKNIN Sophie, AKODAD Hayatte, AKOU'OU Jean-Florent, AL FAOUR Firas, AL WARAGLI Noha, ALAUZY Virginie, ALAZRA Bernard, ALBY Marie-Laure, ALDANDACHI Ghanima, ALETTI Marc, ALEXANDRA Jean-Francois, ALEXANDRE Marie, ALFANDARI Marie-Elise, ALLEZ Matthieu, ALLIEL Celine, ALNASER Feras, ALOUI Khouloud, AMADOR BORRERO Blanca, AMADOR Maria Del Mar, AMANI Johan, AMAR Julien, AMAR KHODJA Safia, AMAR Laurence, AMARSY Rishma, AMET Denis, AMEUR Lydia, AMIEL TAIEB Carline, AMOR CHELIHI Lahoueri, AMOURA Anne, AMOYEL Maxime, AMRANE Amar, AMRAOUI Abdelheq, AMRIOUI Fatima, AMSELLEM Julien, ANDEL AMIEL Audrey, ANDRE Arthur, ANDRE Marie Helene, ANDRE Marie H√©l√®ne, ANDRE Thierry, ANDREOTTI Christophe, ANDRONIKOF Marc, ANGLEJAN CHATILLON Emma, ANXO Lionel, AOUIDAD Iman, APARICIO Caroline, ARIFA Yamina, ARISOY Ersin, ARNOULD Thomas Alain, ARNULF Isabelle, ARONDELLE Jean-Michel, ARROUY Laurence, ARROYO Isabel, ARTIGOU Jean Yves, ARTIGOU Jean-Yves, ARTIS Carolie, ASCIONE Sophia, ASSLO Gabriel, ASSOUN Sandra, ATASSI-DUMONT Marie-Elisabeth, AUBRY Adeline, AUBRY Alexandra, AUDIER-BOURGAIN Marie, AUDIFFRED Laeticia, AUFFREDOU Fran√ßois Pierre, AUGER Martin, AUGER Yvain, AVENEAU Clement, AVONDO Aur√©lie, AVOUAC Jerome, AYLLON MILLA Sonia, AYOUBI Jean-Marc, AZEMAR Laurene, AZOULAY C√©lia, AZRIA Elie, BAARIR-CHELAH Nacera, BACHELLEZ Jordan Nady, BACHIR Dora, BACHMEYER Claude, BADOC Laurianne, BAGNIS Corinne, BAH Mariama, BAILLEUX Anne, BAKER Catherine, BAKIRLI Birol, BAKKOUCH Abderrahmane, BAKOPOULOU Sofia, BALAVOINE St√©phanie, BALLESTER Marie, BALLET Amelie, BALLOUE Claire, BANNEVILLE B√©atrice, BARBEAU Guillaume Paul, BARBEROUX Vincent, BARBIER Olivier, BARD Anne Sophie, BARD Claudine, BARDIN Gilles, BARGHOUT Majed, BARON Marine, BARRAULT Laure, BARRE Amelie, BARRELET Audrey, BARROS DE FREITAS Marta, BARTHIER Sophie, BARTOLUCCI Pablo, BASMACI Romain, BASSE Clemence, BASSIEUX Audrey, BASTIANI Sophie, BATISSE Dominique, BATISTA Cecile, BATONGA Marc, BAUDANT Simon, BAUDOUIN Clemence, BAUDRY Elodie, BAUVOIS Adeline, BAYLAC Pierre, BAYOUB Hasna, BAZIN Thomas, BEAUDEAU Lauren Marylin, BEAUDONNET Guillemette, BEAULIEU D'IVERNOIS Diane, BEAUNE Sebastien, BEDOCK Doroth√©e, BEDOSSA Alexandra, BEGGAZ Yasmine, BEHTASH Ariana, BEITZ Marie, BEKHIT Shana, BEL LASSEN Pierre, BELAN Adrien, BELARBI Linda, BELAUBE Nicolas, BELGHITH Imen, BELHADJ Karim, BELKACEM Anna, BELKHIR Rakiba, BELKHODJA Omar, BELLAICHE Guy, BELLANTONIO Sophie, BELLOC Jeanne, BELLOCQ Agn√®s, BELMIHOUB Ines, BELPOMME Vanessa, BEN ZINA Nader, BENAINOUS Olivier, BENAMOUZIG Robert, BENATTIA Amira, BENAY Cecile, BENAZRA Albert, BENCHETRIT Deborah, BENCHETRIT Kevin, BENCHIMOL Gabriel, BENDETOWICZ David, BENHELLAL Anouar, BENKEMOUN Fanny, BENMAYOUF Thiziri, BENOTHMENE Omayma, BENZADON Judith, BENZAQUEN Helene, BERCHERIE Julie, BERENBAUM Francis, BERGER Ingrid, BERGERON Corinne, BERINGER Juliette, BERLET Sabine, BERLEUR Marie, BERMAN Audrey, BERMUDEZ Julien, BERNARD Charles, BERNARD Sandra, BERREUR Sylvie, BERREZAG Walid, BERTHAUX Lise, BERTHIER Servane, BERTHOL Naima, BERTON Laurence, BESSAC Jean Francois, BESSELERE Renaud, BESSIS Simon, BETARI Btissame, BEURNIER Antoine, BEYDON Maxime, BEYRON Anne, BEZIAUD Frederic, BIALOBRODA Jonathan Ariel, BIBAS Jordan, BIBI NGOYI Natacha, BIDEAULT Herve, BIHOREAU Magali, BIJON PERRET Amelie, BILGAR Antonela, BILGAR Antonella, BILLARD Charlotte, BILLIAUWS Lore, BINETRUY Charles, BITTAN Jerome, BLACHEZ Marion, BLANC Arnaud, BLANCHE Philippe, BLANDIN Camille, BLED Quentin, BLEIBTREU Alexandre, BLIN Emmanuelle, BLOCH-LAINE Emmanuel, BOCHE Thevy, BOCQUET Hadrien, BOISSERAND Luc, BOIZE Julien, BOKOBZA Jerome, BOLIGNANO Lucie, BOLLENS Diane, BOMPARD Rudy Pierre, BONDEELLE Louise, BONELLO Kim, BONGRAND Christophe, BONIER Colombine, BONNAMOUR Beatrix, BONNETON Marion, BORIE Alice, BORIE Constance, BORNES Marie-Isabelle, BOSREDON Clotilde, BOUABDELLI Ikram, BOUALLEGUE Sarah, BOUBKEUR Nasser, BOUBLIL Paul, BOUCHARD Jean-Loup, BOUCHAUD Olivier, BOUCHEZ Saena, BOUCHOUCHA Michel, BOUCHY Nicolas, BOUCLY Athenais, BOUCLY S√©gol√®ne, BOUDALI Yasmina, BOUDCHICHA Boris, BOUDDAHAB Hanane, BOUDERAH Karima, BOUE Fran√ßois, BOUEILH Anna, BOUGUENNA Idriss, BOUHNIK Yoram, BOUIDIDA Bayane, BOUILLET Thierry, BOUKANGA NKOULOU Nina Prisca, BOUKEBOUS Baptiste, BOULATE David, BOULDOUYRE Marie-Anne, BOUNACOEUR Hedi, BOURAS Leila, BOURDIN Nathalie, BOURDIN Vanceslas, BOURGARIT DURAND Anne, BOURGEOIS Antoine, BOURGEOIS Melanie, BOURGON Nicolas, BOURRIER Anne, BOURRON Olivier, BOUSCARY Didier, BOUSSABOUN Donia, BOUSSARIE Lucas, BOUSSAUD Veronique, BOUSSEN Ines, BOUSSION Helene, BOUTONNE Romain, BOUTOT Fran√ßoise, BOUVARD Eric, BOUVATTIER Claire, BOUZIRI Nesrine, BOYER CHAMMARD Timothee, BOYER Jean Baptiste, BOZIC Marjana, BRACHAT Joelle, BRAGANCA Ad√©lia, BRAHIMI Melina, BRANCHEREAU Louise, BRAVAIS Juliette, BRAZILLE Patricia, BREAL Claire, BREDIMAS LEMARIEY Aliki, BRESSAND Sabine, BRETAULT Marion, BREVART Christophe, BRIGANT Fabien, BROSSEAU Solenn, BRUNEAU Catherine, BRUNEAU Marie, BUFFEL DU VAURE C√©line, BUFFET Camille, BUFFO Milene, BUI PHAN Thi Eida, BUKREYEVA Iryna, BULIFON Sophie, BULTEAU Juliette, BUNG Kheravy, BURCIN Cecilia, BURGGRAFF Eric, BURKOVIC Laetitia, BURLACU Ruxandra, BUSSIERES Laurence, BUSSONE Guillaume, BUZZI Jean-Claude, CABON Mathieu, CACOUB Lea, CADENNES Alice, CADIERGUES Delphine, CADOT Angelique, CADRANEL Jacques, CAILHOL Johann, CALLIAU Audrey, CAMARA Ousmane, CAMELLI Scarlett, CAMPA Pauline, CAMUS Baptiste, CAMUS Margaux, CAMUS Marine, CAMUSET Juliette, CANAVAGGIO Pauline, CANCELLA DE ABREU Marta Isabel, CANELLAS Anthony, CANESTRI Ana, CANOU√è Etienne, CANTIN Delphine, CARADEC Emmanuella, CARAUX PAZ Pauline, CARBONNEL Franck, CARETTE Claire, CARILLION Aude, CARLIER Nicolas, CASALINO Enrique, CASEDEVALL Marion, CASELLI Florian, CASERIS Marion, CASSAGNE Angele, CASSARD Clementine, CASSIANIDES Cedric, CASSIANIDES C√©dric, CASSIAUX Audrey, CASSUTO Margot, CAUCHEMEZ Bruno, CAUQUIL Cecile, CAZENAVE Benoit, CECCON Elsa, CELERIER Julie, CERCEAU Remi, CESCON Laure, CHA Olivier, CHAABANE Rim, CHAILLET Muriel, CHAKHTOURA Zeina, CHALEIL Soisic, CHALVIGNAC J√©r√¥me, CHAMBRIN Veronique, CHAMPION Karine, CHANSON Noemie, CHANTALAT AUGER Christelle, CHAOUKI Zouhair, CHAPEAU Romain, CHARARA Oussama, CHARLIER Caroline, CHARNEY Alexandre, CHARPENTIER Camille, CHARY Isabelle, CHAS Julie, CHASSAING Augustin, CHASSOUX-TRUFFINET Francine, CHAUCHARD Maria, CHAUSSAIN Catherine, CHAUVEAU Simon, CHAUVET Sophie, CHAUVIN Anthony, CHAVANNE Denis, CHAY Yves, CHAZAL Thibaud, CHEAIB Sadek, CHEKROUN Malika, CHELOUFI Meryam, CHEMINET Geoffrey, CHENG Charles, CHEOUX Julie, CHERMAK Aziza, CHEVALIER Amelie, CHEVALIER Am√©lie, CHEVALIER Julia, CHEVALIER Pauline, CHIARABINI Thibault, CHINET Thierry, CHOINIER Pascaline, CHOLLET-XEMARD Charlotte, CHOLLIER Antoine, CHOPIN Doroth√©e, CHOQUET Sylvain, CHOSIDOW Anais, CHOUIKH Taieb, CIANGURA Cecile, CIOVICA Gilda-Alina, CISSOKHO Khadidiatou, COGNAT BRAGEOT Mathilde, COGNE Yann, COHEN Fleur, COHEN Frederique, COHEN Laure, COHEN Pascal, COHEN Raphael, COHEN R√©my, COILLY Audrey, COLARD Martin, COLAS Bernard, COLAS Marion, COLLARINO Rocco, COLLET Val√©rie, COLLIN Elisabeth, COLMANT D’ARMAGNAC Claire, COLOMES Abel, COLOSI Luisa, COMARMOND ORTOLI Chloe, COMBA Marianne, COMBES Sabine, COMBET Margot, COMBIER Alice, COMMIEN Laura, COMPARON Celine, CONAN Pierre-Louis, CONTEJEAN Adrien, CONTI Isabelle, COQUARD Marie Charlene, CORCOS Olivier, CORDEL Hugues, CORIAT Romain, CORNU Erika, CORPECHOT Christophe, CORRE Vassili, COSTANTINI Adrien, COSTES Yolaine, COUCHOT Julien, COUEROIS Pauline, COUFFIGNAL Julie, COULIBALY Kadiba, COULOMB Audrey, COURSEAU Romain, COURTIN Thomas, COURVOISIER-BERTRAND Marie, COUSSEMENT Francois, COUTANCEAU Bertrand, COUTANT Aude, COUTURE Priscille, CRAVASSAC Lauren, CRICKX Etienne, CROCHETON Nicolas, CURAC Sonja, CURTIS William, CURY Nicolas, CUVEILLIER Daphnee, CUVEILLIER Gery, DA SILVA CAIADO Tomy, DA SILVA Melissa, DABBAGH Sami, DAFFOS Quentin, DAHAN Karine, DAHDOUH Houaida, DAHMANE Lotfi, DALLE Ludovic, DAMAS Victor, DAMAS-PERRICHET Clara, DANA Jeremy, DANGLES Marie Therese, DANIA-JAMBU Elsa, DARDEL Corinne, DARDENNE Anne Laure, DARMON Arthur, DARMON Emilie, DASKALEROU Markella, DAUTHEVILLE Sandrine, DAVENNE Beatrice, DAVID Steven, DAVIDO Benjamin, DAYAN L√©a, DE BASTARD Laurent, DE BOYSSON Bertrand, DE CARNE Marie-Charlotte, DE CASTELBAJAC Flore, DE CHARRY Felicite, DE COURTEIX Charlotte, DE FREMINVILLE Jean-Baptiste, DE FREMINVILLE Quiterie, DE GOUVELLO Amaury, DE ITTAH BENABOU Corine, DE LA FORTEL Sophie, DE LA FORTELLE Sophie, DE LUNA CARDENAL Gonzalo, DE MALGLAIVE Pauline, DE MARTIN Eleonora, DE MEDEIROS Hugues, DE MENTHON Mathilde, DE MESTIER DU BOURG Louis, DE PALEZIEUX Olivier, DE PARADES Vincent, DE RAIGNIAC Axelle, DE SALINS Victoire, DE TRUCHIS Anne, DE TRUCHIS Pierre, DEAU Segolene, DEBRAY Agathe, DEBRUYNE G√©raud, DECONINCK Laurene, DECROOCQ Justine, DEFOUR Thibault, DEGER Numan, DEKEYSER Manon, DEKIMECHE Ahmed, DELAHAYE Tiphaine, DELAISI Bertrand, DELALEU Jeremie, DELANNOY Quentin, DELANOE Clelia, DELAPORTE DESVAUX Cl√©mentine, DELATTRE Jean-Francois, DELAVAL Laure, DELAVAUX Christine, DELAY Mathieu, DELCEY Veronique, DELERME Samuel, DELFORGE Juliette, DELHOMME Clemence, DELORME C√©cile, DELUNEL Arnaud, DEMARIA Lucie, DEMARICOURT Pierre, DEMEESTER Antoine, DEMORY Nathalie, DENANTES Mady, DENIAU Nicolas, DENIS Blandine, DENIS Christian, DEPIL DUVAL Arnaud, DEPOND Audrey, DEPRET VASSAL Jacqueline, DEREIX Adrien, DEROCHEBRUNE Charlotte, DERRADJI Ouda, DES GARETS DE GARNIER Juliette, DESBANT Aurore, DESCAMPS Elise, DESCHAMPS Claire, DESFEMMES- BALEYTE Tsellina, DESFORGES-BULLET Virginie, DESJARDINS Clement, DESMAIZIERES Michel, DESMAIZIERES Michel Henri, DESPLANQUES Manon, DESTRIBOIS David Israel, DEUTSCH David, DEVALLIERE Emilie, DEVAQUET Niaz, DEVAUX Mathilde, DEVILLERS Magdalena, DEVRED Ines, DEZELEE Remy, DHOOGE Marion, DIAKIT√â Sarah, DIALLO K√©vin, DIANTEILL St√©phanie, DIARD Capucine, DIAZ Maxime, DIB Faiza, DIENG Mouhamed, DIEUDE Philippe, DIEZ Stephane, DIEZ St√©phane, DINH Aur√©lien, DJAAFRI Fatiha, DJAMOURI-MONNORY Fatima, DJEBBAR Yamina, DJIAN Cassandre, DO Ariane, DO CAO Jeremy, DOMAN Marc, DOMIGO Valerie, DOMMERGUES Marie Aliette, DOMONT Fanny, DONNADIEU Dephine, DORENT Richard, DOUEDARI Fadia, DOUGE Guillaume, DOUMENC Benoit, DOURNON Nathalie, DOUVRY Benoit, DRIESSEN Marine, DROGREY Marie, DROUIN Sarah, DUBERNET Tiphaine, DUBERT Marie, DUBOS LASCU Georgeta, DUBOSQ Valerie, DUBOURG GORY Genevi√®ve, DUCHEMANN Boris, DUCHENOY Thibault, DUCLERCQ Chloe, DUCROCQ Celine, DUFAU Romain, DUFEU Virginie, DUFOUR Quentin, DUGUET Theo, DULAC Anne-Sophie, DUMASY Pierre, DUMENIL Coraline, DUMONT Celine, DUMONT Margaux, DUNOGUE Bertrand, DUNOYER Violaine, DUONG Thieu Hung, DUPIN Clairelyne, DUPRE LA TOUR Alexandra, DUPRE-LAILLER Axelle, DUPUIS BERTI Mathilde, DUPUIS Jehan, DUPUY Christiane, DUPUY Olivier, DURANTEAU Lise, DURIEZ-MISE Ga√´le, DURRMEYER Xavier, DUTEURTRE Martin, DUVAL Laure, DUVAL-ARNOULD Marc, DUVOUX Christophe, DYENS Julia, EBADI Vahid, EBOUE Florence, EBSTEIN Esther, ECHARD Alexandre, ED-DEMRI Youssef, EDWARDS Marine, EL GNAOUI Taoufik, EL HUSSEINI Kinan, EL KAROUI Khalil, EL SHARKAWI Chanez, ELDIRANI Charif, ELEZI Arben, ELIAHOU Ludivine, ELLINGER Alexandre, ELLOUZE Sami, ELOI-BLEZES Sonia, EME Anne-Line, EMILE Elsa, EMONT Renaud, EMY Sylvain, ENACHE ANGOULVANT Adela, EPAUD Christelle, ESNAULT Helene, ESTRUCH Manon, ETIENNE Nicolas, EWENCZYK Claire, EYER Xavier, EZANNO Anne Cecile, FABRE Jean-Pierre, FAES Diane, FAIN Olivier, FALL-ARCENS Fatima, FALLET Vincent, FARA Amandine, FARGIER R√©mi, FAUCHER Lise-Noelle, FAURE Christine, FAURE Valentine, FAUTREL Bruno, FAVIER Marion, FAVRE Angeline, FAYAND Antoine, FAZEL Afchine, FEDELI Ilaria, FELLAG CHEBRA Fatima, FERON Florine, FERRAGUTI Aurelia, FEYEUX Delphine, FICKO Cecile, FILIPOVA Paulina, FINET Flora, FIOR Renato, FIRMIN Marine, FLANDRIN Jennifer, FLAUDER Elodie, FLEURET Cl√©ment, FLEURY C√©line, FLIPO Caroline, FOIS Elena Judith, FOLTZ Violaine, FONQUERNIE Laurent, FONTAINE Jean-Paul, FORCE Thibault, FORNASARI Marta, FORTIER Frederique, FORTIN Guillaume, FOTSO FODOUOP Joseph, FOUDI Farid, FOULON GRUMBACH Lorraine, FOURGEAUD Caroline, FOURNIER Marie-Gabrielle, FOURREAU Frederic, FRAIGNEAU Olivia, FRANCHI Patricia, FRANCHITTI Jessica, FRANCINI Veronique, FRANCOIS Helene, FRAPPA Murielle, FRAZIER Aline, FRECHILLA Elodie, FREIRE MARESCA Maresca, FRELAUT Maxime, FREMONT GOUDOT Guillemette, FREY Alain, FREY Carole, FREYNET Olivia, FROC Chlo√©, FROGUEL Eric, FU Shouyu, FUMERY Maxime, FUROIS Caroline, FURTADO LEAL Meg, GABARRA Elisabeth, GABILLARD Zoe, GABILLARD Zo√©, GACEMI Djalila, GALICHON Bertrand, GALICHON Pierre, GALLAND Joris, GALLIEN Sebastien, GALLO Antonio, GALLOT Claire, GALTIER Veronique, GALY Adrien, GANANSIA Olivier, GARANDEAU Elzbieta, GARDERET Laurent, GAREL Bethsabee, GARNIER Clarisse, GARNIER Jean Christophe, GARNIER Virginie, GARREC Nathalie, GARRIQUES Philippe, GASCHIGNARD Jean, GASNIER Karine, GASSAMA Salimata, GATAA Ithar, GATEY Caroline, GAUBE Geraud, GAUDILLAT Christophe, GAUDRON Sophie, GAUTHIER Diane-Cecile, GAUTHIER Eric, GAUTIER Maxime, GAUTRON Marie Astrid, GAY Matthieu, GENEVOIS Marie, GENEVOIS Sandra, GEORGIN LAVIALLE Sophie, GEORGY Hermine, GERIN Magdalena, GERMAIN Dominique, GERVAIS HASENKNOPF Anne, GESTIN St√©phanie, GHOUILES Sabrina, GIANNESINI Claire, GIELY David, GIESI Cl√©mence, GIGANDON Anne, GILBERT Marie, GILBERT Rapha√´l, GILLAS-BURON G√©raldine, GILLE Thomas, GILLERON Pauline, GILLES-REMOND Stephanie, GILLY Alice, GIRALDO BELTRAN Natalia, GIRARD C√©cile, GIRARD Guillaume, GIRARD Paul, GIRAUD Violaine, GIROUD Marion, GIROUX LEPRIEUR Benedicte, GLASMAN Pauline, GLIKMAN Martine, GLOAGUEN Sabrina, GOBERT Delphine, GODARD LALANDE Marie, GODARD Marie, GOEAU-BRISSONNIERE Marc, GOHIER Lucas, GOIX Laurent, GOLDMAN William, GOLETTO Tiphaine, GOLFIER Eve, GOMES Tania, GOMEZ Florie, GOMEZ RODRIGUEZ Eduard, GOMINET Marie, GORLICKI Judith, GOTTLIEB Jeremy, GOUALARD Frederique, GOUAMI Imad, GOUDOT Guillaume, GOUJARD Cecile, GOULENOK Tiphaine, GOULET Helene, GOUNANT Valerie, GOURAUD Francois, GOUTAGNY Stephane, GOUTAL Simon, GOUVERNEUR Capucine, GRABLI David, GRANOTIER Fanny, GRAS Emmanuelle, GRAS Julien, GRASLAND Anne, GRATEAU Gilles, GRAVELLE Pauline, GRAZINA Stephanie, GREFFE Segolene, GREFFET Agnes, GRENET Julie, GRENIER Adrien, GRIDEL H√©l√®ne, GRIFFAIS R√©my, GRIMBERT Michel, GROSS Ariane, GRUNBERG Philippe, GUEDJ Thierry, GUENIN Aur√©lien, GUERICOLAS Maximilien, GUERIF DUBREUCQ Evelyne, GUERIN Capucine, GUESOUM Abdelhamid, GUETTA Michael, GUETTROT IMBERT Gaelle, GUIDDIR Tamazoust, GUIGNAT Laurence, GUILGAUD Francois, GUILLAUD DANIS Constance, GUILLAUME CZITROM Severine, GUILLAUME-JUGNOT Perrine, GUILLET Henri, GUILLET Matthieu, GUILLET Stephanie, GUILLOT Helene, GUILLUY Carole, GUINEMER Sabine, GUITI Chabnam, GULIAN Tim, GUOI Philippe, GUSSI Ilinca, GUTERMAN Sarah, HA Emilie, HAAS Laurent, HACHEMI Amel, HACIL Abdelhakim, HADDAD Zouhair, HADDOUCHE Abdallah, HADJEM Ali, HAGE Mirella, HAGUENAUER Didier, HAINQUE Elodie, HAJOUJI IDRISSI Linda, HAKIM Nicolas, HALBRON GUENANCIA Marine, HALITIM Pierre, HAMMAD Kahina, HAMZA Lilia, HANDSCHUH Richard, HANNANE Souad, HANON Cecile, HANSCONRAD Erwin, HARNOIS Florence, HARTEMANN Agnes, HARTEMANN Agn√®s, HASCOET Sebastien, HASSENFORDER Marie-Pierre, HEFEZ Louise, HEGGARTY Estelle, HEID Beno√Æt, HEINZLEF Olivier, HELLIER Jean Philippe, HENEAU Alice, HENOCQ Sandra, HENRIQUEZ Soledad, HENRY Julien, HENTGEN Veronique, HERMAND Christelle, HERNANDEZ NASS Emilia, HERROU Anne-Flore, HIANCE-DELAHAYE Anne, HIDDEN LUCET Fran√ßoise, HIE Miguel, HIESSE Isabelle, HILBRUNNER Frederique, HILLAIRE Sophie, HILLION Brigitte, HINGLAIS Etienne, HIRSCH Gaelle, HIRSH Sylvain, HODY Julien, HOMS Sebastien, HOMSY Catherine, HONOLD Elodie, HONSEL Vasco, HOOKER Corinne, HORIOT Alexandre, HOUENOU Josselin, HSING Priscilia, HUET Stephanie, HUET St√©phanie, HUOT MAIRE Valerie, HURIEZ Pauline, HUSCENOT Tessa, HYRON Guillaume, IBN SAIED Wafa, IBN-SAID Wafa, IBRAHIM Aymard, IDDAHAMOU Moussa, IGUAL Jeanine, ILLOUZ St√©phane, IMBERT Simonne, IONESCU Roxana, IREKTI Faliha, ISMAEL Sophie, ISSILAME Yasser, IUNG Bernard, JAAFAR Daniella, JABER Samy, JABRE Patricia, JACHIET Vincent, JAEGER Roland, JAFFAL Karim, JAIS Xavier, JAMI Ilan, JANNIC Arnaud, JANUEL Edouard, JAOUEN Lucie, JARROUSSE Bernard, JAULERRY Sarah, JAUREGUIBERRY Stephane, JAZIRI Asma, JEANNE Catherine, JEANNERAT Anne-Gabrielle, JEANNIN Anne-Caroline, JENY Florence, JESTIN Christine, JEVNIKAR Mitja, JINQ M√©lanie, JOBEZ Emmanuel, JOCKEY Christine, JOLLY Pascale, JOLY Charlotte, JOLY Francoise, JOLY Veronique, JONDEAU Guillaume, JOUABLI Moenes, JOUMAA Hassan, JOU√ãT Pauline, JUILLARD Bertrand, JULLA Jean-Baptiste, JULLIOT DE LA MORANDIERE Camille, JUNGELSON Amelie, JUTANT Etienne Marie, JUTANT Etienne-Marie, JUVIN Karine, JUVIN Philippe, KADOUCH Diana, KADRI Sabrina, KAHN Jean Emmanuel, KALAMARIDES Sophie, KAMBOUA Mounir, KANAAN Reem, KANSAO Jamal, KARBASSI Marianne, KARGOUGOU Eric, KARMOCHKINE Marie, KARMOCHKINE Marina, KASSASSEYA Christian, KASSER Camille, KATLAMA Christine, KAYAT Jeremy, KEROMN√àS Gwenola, KETFI Chahinez, KETTANEH Laurence, KEVORKIAN Jean Philippe, KHAU David, KHEBRI Faten, KHELIFAOUI Lysa, KHELLAF Mehdi, KHENISSI Khalil, KHIARI Mehdi, KHOSROWSHAHI Anoosha, KIEFFER Etienne, KIENER Melusine, KIERZEK Gerald, KLEMENT Elise Marie, KLENKLE Laurence, KLERE Isabelle, KLOECKNER Martin, KOLAKOWSKA Agnieszka, KORT Fatma, KOUNG Nancya, KOWALSKI Clotilde, KRIEF Axel, LA MARTIRE Giulia, LABARTHE Amandine, LABEYRIE Celine, LABITRIE Pierre, LABOUCHEIX Annie, LABRUNE Sylvie, LACAILLE Antoine, LACHAUME Noemie, LACOMBE Karine, LACROZE Laurence, LACROZE Marie, LADARRE Christophe, LADKA Agnes, LAFAURIE Matthieu, LAFI Mehdi, LAFOESTE H√©l√®ne, LAFONT Alexandre, LAFONT Emmanuel, LAHAYE Claire, LAKHLIFI Marie-Ines, LAMARRE Philippe, LAMHAUT Lionel, LAMOURIC Christophe, LANCIEN Solenn, LANDMAN C√©cilia, LANDOWSKI Stephanie, LANGLOIS Jessica, LANKAR Mathilde, LAPARRA Ariane, LAPERRELLE Juliette, LARAKEB Skander Anis, LARBI Abdelaziz, LAREDO Mikael, LARIVEN Sylvie, LAROUZEE Elise, LASCAR Justine, LASCAUX CAMETZ Anne Sophie, LASCAUX-CAMETZ Anne-Sophie, LASCOUX COMBE Caroline, LASNE Vincent, LAURENT Louise, LAURENT Lucie, LAURENT Steven, LAVASTE C√©cile, LAVAUD Justine, LAZARETH Helene, LAZARETH Isabelle, LAZARIDOU Ingrid, LAZERGUES Clara, LE BRAS Philippe, LE FORESTIER Nadine, LE HARIVEL DE GONNEVILLE Alix, LE LIEPVRE Helene, LE PENNEC Nolwenne, LE PENNETIER Olivier, LE THAI Huong, LE Thi Hong Loan, LE VAN Fabien, LE VAVASSEUR Benjamin, LE-BARS Michel, LEBACLE Cedric, LEBAIL Gaelle, LEBAS Laurine, LEBEAUX David, LEBEL Josselin, LEBLANC Nathalie, LEBLOND V√©ronique, LECARPENTIER Edouard, LECARPENTIER Eric, LECLERC Catherine, LECLERCQ Mathilde, LECOMTE Charlotte, LECOMTE Fran√ßois, LEFEBVRE Benedicte, LEFEBVRE Marion, LEFEVRE Edouard, LEFEVRE TANTET Delphine, LEFORESTIER Nadine, LEGAY HOANG Lea Anais, LEGAY L√©a, LEGENDRE Antoine, LEGENDRE Elise, LEGENDRE Nicolas, LEGENDRE Paul, LEGROS Lucie, LEGRU-VUILLET Emilie, LEGUEN Pierre, LEHEMBRE Estelle, LEJEUNE Celine, LEJEUNE Pauline, LEKHAL C√©line, LEKOUARA Daniel, LELEU Agathe, LEMARIE Nadege, LEMPEREUR DE GUERNY Marie Claude, LENGLET Tmothee, LENOIR Gilles, LEO Schlaefflin, LEONARD COLLADO Arthur, LEPAVEC Jerome, LEPELLETIER Mathilde, LEPEULE Raphael, LEPRIS√â Olivier, LEROLLE Nathalie, LEROUX Claire, LEROUX Ga√´lle, LEROY Pierre, LESCURE Xavier, LETAVERNIER Emmanuel, LETELLIER Alice, LETEMBET-IPPET Valerie-Anne, LEUNG Ines, LEVASSEUR Anne Sophie, LEVECQUE Karine, LEVY Dora, LHEIMEUR Camille, LHEURE Coralie, LHUISSIER Fran√ßois, LIEGEON Geoffroy, LIEVRE Thomas, LILAMAND Matthieu, LIMAL Nicolas, LINGLART Agnes, LIOTE Frederic, LOEB Thomas, LOEUNG Hoang Uyen, LOIZEAU Maxime, LONDON Jonathan, LOPES Amanda, LOPES Anne-Sophie, LOPEZ SUBLET Marilucy, LORILLON Gwenael, LORROT Mathie, LORUT Christine, LOSCHEIDERA Anne Marie, LOYER Guirec, LUCAS AMICHI Axelle, LUCENAY Sonia, LUK SAONA Helen, LUSTMAN Caroline, LY Axelle, LY Benoit, LY CONG Philippe, LY Reaksmei, M SILTI Linda, MA Sabrina, MAAREK Alizee, MACAIGNE Gilles, MACHADO Moise, MADHI Fouad, MADJLESSI Arach, MADOUGOU SARIKI Aida, MAEVAS Thibault, MAGEAU Arthur, MAHADEVAN Pranesh, MAHEVAS Matthieu, MAHMOUDI Said, MAISONNEUVE Emeline, MAJDLING Alena, MALAK Sandra, MALGRAS Brice, MALLAT Ariane, MALLERET Lea, MALLERET L√©a, MALOT Claire, MALRIN Roxane, MAMONA KILU Christel, MAMPOUYA Davy, MANAMANI Jaffar, MANASTERSKI Fabien, MANDA Victoria, MANIVIT-LALOUM Anais, MANSENCAL Nicolas, MANSOURI Nadia, MANSOURI Sana, MAO Celina, MARBEUF-GUEYE Carole, MARCAIS Ambroise, MARCHESI Laura, MARCIANO Eva, MARCIANO Julie, MAREUSE Segolene, MARIE AUDE Penet, MARIJON H√©l√®ne, MARINEAU Maryvonne, MARTHE Axelle, MARTIN Anne-Celine, MARTIN Auger, MARTIN Camille, MARTIN DE VAUXMORET Claire, MARTIN Lucille, MARTIN Yann, MARTINS-GONCALVES Filipa, MARZOUK Nessrine, MASBOU Helene, MASCITTI Helene Tiphaine, MASOT Emmanuel, MASSARI Anne, MASSON Helene, MASSOT Julien, MASS√â Pascale, MATHEVET Thierry, MATHIEU Geraud, MATT Morgan, MATTIO Clemence, MATTIONI Sarah, MATTON Lise, MAZOUZ Sihem, MAZOWIECKI Maxime, MBEDE Yves, MBESSE Marie-Laurence, MCFADDEN Michael, MECHAI Frederic, MECHAI Soufiane, MEDINA Fernanda, MEHAWEJ Hanane, MEILHAUD Marie France, MEJEAN Elodie, MEKACHER Hamida, MEKINIAN Arsene, MELICA Giovanna, MELOT B√©n√©dicte, MENARD Anne-Marie, MENERET Aurelie, MENVIELLE Camille, MERAZGA Sihem, MERAZKA Amel, MERCADAL Lucile, MERCADIER-RIAZ Elise, MERCIER Olaf, MERZOUK Aicha, MESNIL Celine, MESPLEDE Caroline, MESSALI Anne, MESSIKA Jonathan, MEUNIER Emilie, MEUNIER Marine, MEWASING Baboo Irwinsingh, MEYNIEUX Ulysse, MEYSSONNIER Vanina, MEZIANE Sarrah, MICHELIN Marie, MICHON Adrien, MIGNARD Xavier, MILCENT Karen, MILLET Floriane, MILLOT HATT Claire, MINKA Fadi Hillary, MION Mathieu, MIR Chadia, MIR Claudia, MIRAULT Tristan, MISSUD David, MOGHADDAM Rebecca, MOGNON Kore, MOINARD Charlotte, MOKRI Anais, MOLINA Jean Michel, MOLINA Jean-Michel, MOLITOR Jean Luc, MOLLIERE Chloe, MONCELLY Laurence, MONNET Claire-Marie, MONNET Margaux, MONNIER S√©bastien, MONSEL Stephanie, MONTAGLIANI Lionel, MONTAGNON Sophie, MONTANI David, MONTOUT Anne-Lise, MORACCHINI Lucile, MORARDET Laetitia, MORBIEU Caroline, MOREAU Juliette, MOREAU Pauline, MOREL Cl√©mence, MORER Lise, MORGAND Marjolaine, MORGANT Stephanie, MORIZE Veronique, MORNAND Pierre, MOSTEFAI-DULAC Yasmine, MOUAS Houria, MOUDOUD Rachid, MOUGEOT Christine, MOULAY Khira, MOULIN Florence, MOULY Stephane, MOURAD Jean Jacques, MOURAD Jerome, MOURAD J√©r√¥me, MOUREN Domitille, MOUREY Pascaline, MOUTON Claire, MOUZAOUI Mourad, MULLER Stephanie, MUNIER Anne-Lise, MUNOS Nicolas, MURET Alexandre, MUSSET Caroline, MUSSOT Sacha, MUSTAFIC Hazrije, MYARA Fran√ßois, NABET Luc, NACHEF Clement, NADAL Marine, NAIT-IGHIL Lella, NAJAFI Aida, NAKAD Lionel, NANAI Oussama, NAPOLI Aurelien, NAPOLI Aur√©lien, NAPPO Sandrine, NATAF Francois, NAY Laure, NEMNI Ariane, NEOUZE Angele, NERAAL Stefan, NGAKO DEUGOUE Alfred, NGALULAMUKENGESHAYI Guy, NGUYEN Alexandre, NGUYEN Antoine, NGUYEN Caroline, NGUYEN David, NGUYEN KHAC Florence, NGUYEN Remy Quang Tuan, NGUYEN THE Lydie, NGUYEN Thu Huyen, NGUYEN Yann, NGUYEN-TH√â Lydie, NHAN Pascal, NICAISE Flora, NICOL Claire, NICOLAS Marie, NIKPAY Farah, NION LARMURIER Isabelle, NITENBERG Kiyoko, NOBILEAU Constance, NOCTURNE Gaetane, NOEL Florent, NOEL Nicolas, NORET Aur√©lien, NOT Adeline, NOUGAR√àGE Pascal, NSER Naima, NUNES Hilario, NUSSBAUM Camille, OBERT Julie, OBOLO Alix, ODI√àVRE-MONTANI√â Marie-Helene, OGEREAU Carl, OHANA Benjamin, ONG You Yong, ONISZCZUK Julie, ORIEZ Constance, ORRIERE Thibault, ORSINI Jean Pierre, OTTAVIANI Sebastien, OUAZENE Zineb, OUEDRAOGO Elise, OUFKIR Majida, OULED Nora, OZENNE Violaine, PACANOWSKI Jerome, PACELLI Johan, PACI Laure, PADOVANO Ilaria, PAILLOT Beatrix, PAINDAVEINE B√©n√©dicte, PAK Alireza, PAMFIL Ruxandra, PANAYOTOPOULOS Virginie, PANCHBHAYA Nabilah, PAOLI Charles, PAQUET Anne-Laure, PARET Didier, PARIENTE Aaron, PARISEY Marion, PARROT Antoine, PARTOUCHE Bethsabee, PATERON Dominique, PATOUILLET Marie, PAUGAM Marie, PAUL Rajneesh, PAVAGEAU Laure, PAVIE Juliette, PAVY St√©phan, PECH Georges, PECHARD Loan, PELAT Marion, PELLET Elodie, PENET Marie Aude, PENTIER Camille, PEPIN Marion, PERCIE DU SERT Marie France, PEREIRA Denis, PEREIRA Laurent, PEREZ Carla, PERIANES PAJARES Carmen, PERNET Julie, PERRET Marie-Raphaelle, PERROD Aur√©lien, PERRONNE Veronique, PERROTIN RAUFASTE Catherine, PERRUCHE Franck, PETIT Alicia, PETIT HOANG Camille, PETIT-AUBERT Arnaud, PETIT-AUBERT Gwenaelle, PEULIER-MAITRE Elora, PEVIRERI Florence, PEYRONY Olivier, PHAM Anaelle, PHAN Aurelie, PHAN Franck, PHILIPPON Anne-Laure, PHLIPPOTEAU Claire, PIALOUX Gilles, PICARD Herv√©, PIETRASZ Daniel, PIGNE Etienne, PIGNON Carole, PIMENTEL Ana, PINOT Jerome, PINTADO Claire, PIOCH Camille, PIRON Prescillia, PITTI Lionel, PLAISANCE Robin, PLANCHAIS Cl√©mence, POEY Nora, POIRIER Elodie, POL Christine, POMEL Anne-Laure, PONS Romane, PONSCARME Diane, POPELIER M, POPELIER Marc, PORCHER Nicolas, POTENCIER Claire, POUCHAIN Aurelie, POUCHELON Clara, POUJOL Vincent, POUJOL-ROBERT Armelle, POULETTY Mary, POURCHOT Diane, POURQUERY Marina, POUX Margot, POUYET Victorine, PRADELLE Mylene, PRAT Frederic, PREBET Coralie, PREDA Mariana, PRINCIPE Alessandra, PROCOPI Androula, PUEYO Maria, QUENNEVAT Philippe, QUERTAINMONT Yann, QUI Ten-Ten, RACY Emmanuel, RADTCHENKO Andrei, RAFFETIN Alice, RAFFIN-SANSON Marie-Laure, RAIMOND Florence, RAKOTOARISOA Jenny, RAMBAULT Charlotte, RAMTOHUL Ramessur, RANAWANA Sujee, RANAWANA Sujeewa, RANDRIANANDRASANA Teandraza, RANERISON Roger, RANQUE Brigitte, RAPHAEL Maurice, RAQUILLET Claire, RASMUSSEN Camille, RASTOIN Anna, RATNAM Tulasiga, RAVASSE Perrine, RAVET Nathalie, RAYNAL Pierre-Alexis, RAZAZI David, RECH C√©lia, RECH Jean Simon, RECHATIN Bruno, REFABERT Luc, REGARD Lucile, REGNIER Stephanie, REISS Benedikta, REKIK Badreddine, RENAUD Bertrand, RENOU Maxime, REUBRECHT Vanessa, REVAUX Francois, REVUE Eric, RICARD Laure, RICHARD Arnaud Fran√ßois, RICHARD Marie Caroline, RICHARD Marie-Caroline, RICHAUD Clemence, RICHAUD Johanne, RICHIER Quentin, RICHY Cl√©mence, RIGOLET Aude, RIOM Isabelle, RIOUX Christophe, RIPS Nicolas, RISCHARD Julien, RIVIERE Clementine, RIVIERE Cl√©mentine, RIVI√àRE Sebastien, RIZET Valentine, ROBERT Damien, ROBIN Laurence, ROBIN Marie, ROCHE Marie, RODA Nathalina, RODEN ANDERSON Marguerite, ROGER Anissa, ROGER Fran√ßois, ROKICINSKI Mariusz, ROLLAND - SANTANA Pascale, ROLLAND DEBORD Camille, ROMAIN Anne-Sophie, ROMAIN Delphine, RONDEAU Paul, ROORYCK Francois Xavier, ROOS Caroline, ROSE Camille, ROSSARIE Rapha√´le, ROSSO Charlotte, ROTHENBUHLER Anya, ROTNEMER Tamara, ROUCHE Jessica, ROUE Estelle, ROUET Catherine, ROUGES Francois, ROULIN Louise, ROUMI Arnaud, ROUMIER Mathilde, ROUSSEAU G√©raldine, ROUSSEAUX Laetitia, ROUSSEL SIMONIN Cyril, ROUSTANG Isabelle, ROUVEIX NORDON Elisabeth, ROUVI√àRE Nathalie, ROUX Fabienne, ROUZAUD Diane, ROY Jean-Michel, ROY Xavier, ROZENBAUM Anne, ROZENBERG Sylvie, ROZENSZTAJN Nathalie, RUBENSTEIN Emma, RUPPERT Anne Marie, SAAD Mostafa, SAADA Noemie, SAADI Alexandre, SABATE Jean Marc, SABET Sheila, SABOUNDJI Karima, SAFARI Jean, SAGOT Julien, SAGOT Pascal, SAHLI Firaz, SAIDI Khaled, SAIE Clotilde, SAINT-JACQUES Camille, SAKAROVITCH Barbara, SAKHRI Khalil, SALAKOS Stavroula Eleonora, SALDMANN Frederic, SALHI Ahmed, SALHI Nawal, SALINES Jessica, SALMON Dominique, SALOMON Laurent, SALPIN Mathilde, SAMAMA Diane, SANCHEZ Olivier, SANSON Gabriel, SANTIN Aline, SARFATI Julie, SARFATI Samuel, SARKIS AINTABLIAN Gabriel, SASSENOU Jeanne, SAUSSEREAU Julien, SAUVAGET Pascale, SAUVAGNAC Rebecca, SAVALE Laurent, SAVEY Lea, SAWICKI Benedicte, SAWICKI B√©n√©dicte, SAYIN Anne-Sara, SBEIH Nabiha, SBISSA Yannis, SCAIN Anne-Laure, SCEMAMA Agathe, SCETBUN Elsa, SCHEIWE Cedric, SCHERNBERG Antoine, SCHLEMMER Frederic, SCHMIDT Julien, SCHMIDT Madeline, SCHNEIDER Luminata, SCHULER Julie, SCHURR Benjamin, SCHWARTZ Mathieu, SEANG Sophie, SEFERIAN Andrei Horia, SEGOUIN Christophe, SEGUIER Jean-Christophe, SEHIMI Fatima, SELLIER Pierre-Olivier, SEMATI Yamina, SENARD Olivia, SENDE Jean, SENDJAK HARCHE Amina, SENE Damien, SENE Thomas, SENEGAS Manon, SEPTIER Mathilde, SERGEANT Camille, SEROR Raphaele, SERRANO Charlotte, SESE Lucile, SESTITO Estelle, SHI Coralie, SIAVELLIS Justine, SIBI-DIAKITE Dieyanaba, SIBIUDE Jeanne, SIERRA Charlotte, SIFFERT Marc, SIGUIER Martin, SILAR Camille, SIMEON Soline, SIMISDEAN Ciprian, SIMONETTA Marie, SIMONPOLI Anne Marie, SIMONPOLI Anne-Marie, SIOUA Sana, SITBON Olivier, SKENDI Mariela, SLAMA Dorsaf, SLAMA Laurence, SMADJA Laura, SOBOTKA Jennifer, SOKOL Harry, SOLETCHNIK Denis, SOLIMAN Heithem, SONNETTE Agnes, SORBETS Emmanuel, SOUALA-CHALET Yasmine, SOUARE Issa, SOUCHON Lea, SOUCHON L√©a, SOUDAN Damien, SOUDRE Georges, SOUPIZET Francois, SROUSSI Jeremy, STAMBOUL Sarah, STANKOFF Bruno, STEFANESCU Carmen, STEHELIN-GAUDILLAT Catherine, STEICHEN Olivier, STELIANIDES Sandrine, STEPHAN Rolz, STERPU Raluca, STIRNEMANN Julien, STOCCO Cecile, STRAZZULLA David, STROHMENGER Lucas, SUAREZ Jennyfer, SUBRAN Benjamin, SUHL Jaehyo, SULIMOVIC Steeve, SURDON Bartholome, TABET GABISON Alexandre, TABOULET Pierre, TABY Sarah, TAGAWA Samuel, TAIEB David, TAILLE Camille, TAN Soline, TANG Julie, TANGTAKOUN Annie, TANTET Claire, TATIN Marion, TAUBY Gabrielle, TAYBALY Maxime, TAZI Abdellatif, TCHAPI Agathe, TE Nathalie, TEBANO Gianpiero, TEBOUL Alexandre, TECHER Laura, TELION Caroline, TENENBAUM Lisa Perl, TERRIER Sabine, THERBY Audrey, THIEBAUD Pierre-Clement, THIOLLIER Daniel, THIRION Raphael, THOMAS BEAULIEU Domitille, THOMAS Claire, THOMAS Marion, THOUVENOT Aude, TIEU Ashley, TIMBELY Boubacar, TIXIER Denis, TOMA Andrea, TOMASZEK Valentine, TOMEO Charlotte, TOTEL Floriane, TOUAM Maya, TOUATI Elia-Belle, TOUATI Nizar, TOUATI Sylvie, TOUBIANA Julie, TOUMI Houda, TOURNIER Amelie, TOURNON Clement, TOURRET Jerome, TRAVERS Guilhem, TRAVERS Nicolas, TRAVERT Florence, TRECA Pauline, TRETON Xavier, TRIALOUP Sarah Laure, TRIBOULET Felicien, TRICON Apolline, TRINH Fanny, TRINQUE Baptiste, TROUDE Penelope, TROUILLOUD Isabelle, TRUFFIER Ariane, TRUONG Ngoc Vy, TRYSTRAM Noemie, TUNESI Simone, TURC Guillaume, TURPIN Elena, UNGUREANU Aur√©lien, URSU BILLOT Renata, USUBILLAGA Raphael, UZUNHAN Yurdagul, UZZAN Mathieu, VACARU Mariana, VAITTINADA AYAR Prabakar, VALANTIN Marc Antoine, VALCKE-BROSSOLLET Judith, VALENTIAN Maelle, VALERY Solene, VALIN Nadia, VALINVALIN Gilles, VALLEE Fabrice, VALLI Vanina, VALLOT Sophie, VANNIER Mathilde, VANOYE Marie, VANSTEENE Cl√©ment, VATIER Camille, VAUX Julien, VEDRENNE Geraldine, VELLY Laetitia, VERGER Elvina, VERGNEAULT H√©l√®ne, VERNEY Charles, VERNIER Christian, VERNISSE Chrystelle, VERRAT Anne, VERRET GALCERAN Sophie, VERSINI Edouard, VETILLARD Pauline, VEVER Ursulla, VEYLON Pauline, VIARD Jean Paul, VIARD Jean-Paul, VIBERT Flora, VICART Savine, VICIANA Marie, VIDAL Francois, VIGNAUD Olivier, VIGNES Dorothee, VIGNON Marguerite, VIGOUROUX Agathe Laure, VILAINE Eve, VILLEMANT Agnes, VINAS Florent, VINCENT CASSY Christophe, VINDRIOS William, VINIT Caroline, VIRALLY Jer√¥me, VIRONE Alexandre, VITTORIANI Aurelie, VIVANTI Alexandre, VLEDOUTS Serafima, VLEDUTS Seraphima, VODOVAR Dominique, VOISIN Frederic, VULLIERME Marie-Pierre, VUTHY Sy, WAFO Estelle, WALDMANN Victor, WANG Florence, WANNEGUE Laura, WARZOCHA Ursula, WASSERMANN Johanna, WATTIEZ Barbara, WEHBI Samer, WEIL Alice, WEISS Laurence, WEISS Nicolas, WEISSER Isabelle, WEMMERT Charlotte, WEYER Claire Marie, WILLEMS Lise, WISSA Jean Louis, WOIMANT Henri, WOIMANT Maguy, WORMSER Eleonore, WORMSER El√©onore, WU Sang Song, XU Pei, YAMEOGO NABONS WINDE Jean, YASRI Na√Øma, YEFSAH Sofiane, YGER Marion, YIRIK Razal, YORDANOV Youri, YOUNSI Karim, ZAHARIA Ramona, ZAIDAN Mohamad, ZAK Cathia, ZAKHOUR STEPHAN Rola, ZAKINE Adrien, ZAMBON Bernadette, ZANKER Bertrand, ZANKER Caroline, ZAYENE Houcine, ZEARAMANE Souganya, ZEGGAGH Jeremy, ZELEK Laurent, ZENS Claire, ZEPHIR Sol√®ne, ZERROUKI Heidy, ZIANI Mohamed, ZIDI Lamine, ZIEDAN Kamal, ZIENKIEWICZ Matthieu, ZNATY Marie-Laure, ZOUZOUO Marie, ZROUNBA Mathilde Anouche, ZUBER Mathieu, ZUMELZU Coralie, EAP Delphine, ECALE Hortense, ECLANCHER William, ECOLLAN Marie, EKEN Aysun, EKPE Claire, EL AOUD-ABIDA Jouda, EL ASRI Ciham, EL FANI Helene, ELBAZ Sara, ELBEZ Guy, EM-NGOURN Malyda, ENGUEHARD Genevieve, ENGUEHARD Veronique, ENTRESSENGLE Helene, EPEE Geraldine, ERARD Jeanne, ERCOLE Marie-Lou, ESCOBEDO Patricia, ESCOURROU Pierre, ESSER Mickael, ESTADIEU Christine, ESTEGASSY Oury, ETCHEVERRY Marie-Pierre, ETERSTEIN Pierre, ETIENNE Anahi-Laure, EVEN Guy, EVETTE Pascal, FABRE DE MORLHON Olivier, FABY Jacques, FADDA Gaelle, FADDEEFF Lydia, FAGES Sophie, FALCOFF Hector, FAMILY Delphine, FAMILY Shahine, FANTINI Patricia, FARZIN Alain, FAURE David, FAUVEAU Elodie, FAVRE Laury, FAVRE Madeleine, FAVREAU Thierry, FAYE Luc, FELTAIN Pierre, FERRAT Emilie, FERRON Laurence, FERRY Magali, FESNEAU-DEZ Carole, FEVE Morgane, FISCHER Claude, FISCHER Eric, FISCHER Evelyne, FITOUSSI Karine, FLAGEUL Chloe, FLATET Christophe, FLORADIS Kalliopi, FLORENT Nicole, FLORES Corinne, FLOUQUET Marine, FOGIEL Veronique, FOLLIOT Evelyne, FORNIELES Alain, FOUCARD Fanny, FOUGERE Gaelle, FOULT Capucine, FOUQUERE Adrien, FOURMY Laure, FOURNIER Celia, FOY Baudouin, FRANCO Jennifer, FRANCOIS HAUGRIN Lea, FRANCOIS Marc-Aurele, FRANCOIS Mathilde, FRAYRET MARTIN Camille, FREUND-DORISON Veronique, FREYDT Catherine, FRIANT Celine, FRITZ Pierre, FRUGIER Jean-Claude, FURGE Camille, GAGNEUX Christine, GAISNE-JOLY Marine, GALICHON Claire, GALLULA Sebastien, GANDIOL Jerome, GANS Thais, GARCIA Thomas, GARCIA-RIVERO Yilliam, GARNIER Isabelle, GAROT Christelle, GARRIGUES Anne, GARSIN Michel, GARSON Jean-Luc, GASCOIN Jean-Claude, GASGANIAS-ASSAYAG Renee, GASMAN Geraldine, GASPAR Carlos, GAUD-LISTRAT Veronique, GAUFFIER Nora, GAUTHEROT Philippe, GAUTHIER Lauriane, GAUTHIER Raphael, GAY Gabrielle, GAYRAUD Xavier, GAZANIOL Claire, GEANTY Xavier, GEERAERT Jean-Pierre, GELBARD Sophie, GENDREAU-PATROIS Benedicte, GENEIX Nadia, GENELLE Marlene, GENESTE Thomas, GENET Anne, GENET Vivien, GENOT Corinne, GENOUD-TERNAY Elodie, GENTAUD-DAWIDOWICZ Marie-Jose, GENTY-KOLASA Julie, GERARD Baptiste, GERARD Laura, GERAUD Pascal, GERMAIN Christine, GERS Monique, GERVAISOT Jean-Paul, GESQUIERE Anne, GHANEM Sonia, GHAOUI Pascale, GHASAROSSIAN Christian, GHNASSIA Yossi, GHOMARI Asma, GIANG PHAN David, GIANNAKA Dimitra, GIANNOTTI Agnes, GIESZ Jean-Pierre, GIET Eric, GIL Isabelle, GILLES Vincent, GILLET Adeline, GINESTE Anne-Marie, GIRARDON-GRICHY Dominique, GIREY Pierre-Herve, GIRIER Bernard, GIRIN Fanny, GIROLAMI Catherine, GISPERT Jeanne, GODEBERGE Philippe, GOEMANS Armelle, GOGUE MEUNIER Bernard, GOLASIU Aurora Iulia, GOLDKIND Alain, GOLDSTEIN Corinne, GOLDZAK Miryam, GOLFIER Jean-Baptiste, GOLUBOVIC Branko, GORETZKY Boris, GORTCHAKOFF Michel, GOSSET Daniel, GOTTHEFF-SOUSSAN Keren, GOUBY Caroline, GOUDIER Pierre, GOY Pascal, GOZLAN Yohan, GRALL Pascal, GRANDE Roxane, GRANDIN DE L'EPREVIER Marianne, GRANDPIERRE Caroline, GRANERO Gilles, GRANGE-CABANE Armelle, GRASLAND Dominique, GRAVIER Dominique, GREGOIRE Estelle, GRIBI Dacine, GRIDINA Inna, GRIFFON Elise, GRIGNON Clarisse, GRIMAUD Bernard, GRIMAUD Helene, GROELL Nicolas, GRONDIN Olivier, GROSSE Jean Yves, GROUHEL Claire, GRUBER-HUE Sophie, GRUDET Stephane, GRYB Catherine, GUEDJ Pierre, GUEGAN Delphine, GUEGUEN Charlotte, GUENIN Frederic, GUEPRATTE Aurelia, GUERIN Claire, GUERIN Stephanie, GUETTA Liath, GUETTAF Nouadghe, GUEUTIN Thierry, GUEVILLE Christophe, GUIGUI Pascal, GUILBERT Laurence, GUILLAUD Marie, GUILLAUME Clemence, GUILLEMAIN Camille, GUILLON Jeremie, GUILLOSSOU Juliette, GUILLOT Charles, GUILLOT Jean, GUIMARD Gilles, GUIOT-DUCARNE Emeline, GUIRIMAND Marianne, GUITTARD Deborah, GUITTON Jean-Yves, GUITTON Michele, GUTH Pierre-Christian, GUTHMANN Noemie, GUYOT Francis, HABAY Regine, HABERGRYTZ Jean Michel, HABIBI ROUDSARI Pouya, HABOUBI Line, HABRI Nadia, HADDAB Sonia, HADDAD Audrey, HADDAD Fabienne, HADZIC Aida, HALIMI Frank, HAMEURY Stephanie, HAMI Zina, HAMID Malika, HAMM-LEHNER Francoise, HAMOUDI Kamel, HAOUARI Djamila-Ilham, HAR Cecile, HARDY TINARD Sylvie, HARIF Marie, HASSAN Daniel, HAYANI BEN BERKANE Latifa, HAYE-NGUYEN CAO Anne Claire, HAYI-SLAYMAN Hafiz, HAZEN Richard, HELLIER Jean-Philippe, HENG-MOUNIE Kim, HERBAUT Dominique, HERIL Nadia, HERMANT Sandrine, HERVAULT Amelie, HERVIEUX Aurelie, HEYRAUD Philippe, HGUIG Hajar, HILAL Yassine, HIRAUX Emmanuel, HLAVACEK Sabrina, HO A CHUCK Claude, HO-NGUYEN Minh-Ha, HOCQUEMILLER Raphael, HOFFMANN Clara, HOGUIN Jean-Christophe, HOLLIEZ Frederique, HOLLMAN-LEVY Bernadette, HOMASSEL Anne, HOMASSEL Astrid, HOUDART Philippe, HOUSSAINT Christophe, HOUTA Benjamin, HOZE Chloe, HUA Christine, HUA Georges, HUET Christian, HUGEL Stephane, HUGUEL Herve, HUMBLOT-BAYARD Valerie, HUREAU Jean Philippe, HUREL Sophie, HURTAUD Nathalie, HUSSON Frederique, HUSSON Sebastien, HUYNH Jean, HUZER Christine, IBEGAZENE Ali, IGLICKI Franck, IGOUDJILENE Anis, ILLOUZ Stephane, IMBERT Charlotte, INAOUI-ROZE Rachida, INGLESE-ROUX Marie, IRAQUI HOUSSAINI Ilham, ISLA Y ORTIZ Grace, ISRAEL Jonathan, ISSAD Nadia, ITZINGER Philippe, IZADIFAR Armine, JACOB Jean-Pierre, JACQUETY Emmanuel, JACQUIER Vincent, JALADIEU Marie-Helene, JAMIL Jamila, JAMMERON Veronique, JANODY Valerie, JAQUES Justine, JARDY GENTON Carole, JARRY Pierre, JASKIEROWICZ Alain, JAUPI Ilma, JAURY Philippe, JEAN Nathalie, JEANNEAU Laurence, JEANSON Eric, JEANTY Julien, JEOUIT Yamina, JEROME Sylvie, JEUNET Philippe, JHUMMUN Moniza, JIBARD Annaick, JOB Emmanuel, JOBERT Marie, JOBERT-RUFF Claude, JOBEZ Emmanuel, JOLLY-GUYOT Philippe, JOLY Benedicte, JOLY Serge, JON Alice, JONVEL Bruno, JOSSE Philippe, JOUAN Florence, JOUANNAIS Philippe, JOUBERT Marie-Caroline, JOUENNE-ROCHER Julie, JOUIN Christine, JOYEUX CHALLES Pascale, JUBIEN Brigitte, JULIEN-LABRUYERE Delphine, JUNES Eric-Alain, JUSSMANN Sarah, JUSTAUME Gwaenael, KABLA Marine, KACI MAHAMMED CHIROL Lamia, KADOCHE David, KADRI Leila, KALFA Ouriel, KANOUN-DRIRA Hajer, KARAM Sylva, KARAMI Ahmed, KARMAN-MIEL Francoise, KARSINTI Isabelle, KASBI Alexandre, KATZ Christel, KELLERSON Anne, KEMICHE Amina, KENIGSBERG Margot, KERMORGANT Charlotte, KERNER Laurent, KERSUZAN Yves, KERYER-DEGAEY Nathalie, KHALED Rafik, KHALED Salim, KHALFALLAOUI Chloe, KHALLOULI-YAHIA Olfa, KHAZMIM Laura, KHELIL Nacira, KHEYR-POUR Dorna, KHIN Jean-Louis, KIASSI MBAKI Irielle, KICHILOV Lise, KIMVA Lucien, KIRRMANN Agnes, KITOUNI Karim, KIZILIAN Melinee, KLEPPER Caroline, KOIN-MAGANA Nathalie, KONDO Takeshi, KOPP Pauline, KRISHNAKUMAR Jegatha, KUNSTMANN Nicolas, KUPERMINC Martine, L'HUILLIER Jean-Pierre, LABERGERE Thierry, LACANT Francois, LACHAUSSEE Frederic, LACHKAR Etty, LACHKAR Regis, LADAUGE Fabien, LAFARGOUETTE Thierry, LAFFITTE-GOBERT Sandrine, LAFFONT Chantal, LAFORTUNE Jean, LAGHMARI Najib, LAGRANGE Karine, LAINE Luc-Henri, LAIZE Helene, LALANDE Martine, LALUQUE Linda, LAM Man-Chun, LAMACHE Pierre, LAMBERT Frederic, LAMBERT Helene, LAMBERT-BENSIMON Yael, LAMBERT-MONTANI Beatrice, LAMBERTI TEILLET Margherita, LAMBLOT Jean-Luc, LAMHAUT Veronique, LAMY Beatrice, LANCELOT-SOLLOGOUB Anne, LANDIER Elise, LANDRY Catherine, LANDRY-CHASSOT Annyck, LANG Marie-Pierre, LANGLOIS Lena, LANGLOIS Marianne, LAOUANI Hamda, LAOUI Kaci, LAPERRELLE Juliette, LARANCE Eliana, LARGET Dominique, LARNICOL Helene, LAROCHE Margaux, LAROCHE-FICCA Marie-Laure, LASNIER Emmanuelle, LASRY Lauren, LASRY Stephane, LAUCHER Andre, LAUCHER Blandine, LAURAIN Anne, LAURENS Gerard, LAURENT Christophe, LAURENT Eric, LAUVRAY-BOUILLET Caroline, LAVEAU Florent, LAVERGNE Franck, LAZIMI Nadine, LE BIHAN Catherine, LE BOZEC Jerome, LE BRIS Herve, LE CLAINCHE Francois, LE COHU Alain, LE CORRE Patrick, LE DUC-SOLUS Marine, LE FUR Sophie, LE GALL Gaelle, LE GO-DIOSES Nora Maria, LE HENAFF Aude, LE JOUBIOUX Clemence, LE MASURIER Cecile, LE METAYER Charlene, LE MOULT Dominique, LE PRE Annabel, LE TAILLANDIER DE GABORY Brice, LE TRUNG Tu, LEBARON Aurore, LEBEAU Veronique, LEBOEUF-TRAN Celine, LEBOULANGER Helene, LEBOURG Elise, LECALLIER Bertrand, LECART Sylvie, LECAT Genevieve, LECERF Patricia, LECESNE Laure, LECLUSE Nadia, LECOCQ Marlene, LECOCQ Michel, LECOQ Nathalie, LEFEBVRE Didier, LEFEBVRE Stephane, LEFEVRE Antoine, LEFEVRE Karine, LEGAGNEUX Raphael, LEGER Elsa, LEGRAIN Yann, LEI Jing, LEIGNADIER Sylvie, LELEU Catherine, LELONG-BONTE Caroline, LELOU Thi-Anh-Dao, LEMARIEY-BARRAUD Michel, LEMASSON Denis, LEMIERE Pascal, LEMIRE Marie-Christine, LENGIGNON Nathalie, LENOIR-DEBRU Nadine, LEON Saul, LEONARD Laetitia, LEONARD-FAURE Dominique, LEPRETTE Vincent, LEROUGE Jordane, LEROUX Pierre, LEROY Emmanuelle, LEROY Gilles, LEROY Isabelle, LEROY Laetitia, LEROY Lucie, LESAFFRE Xavier, LESAGE Guillaume, LESAGE Isabelle, LESALLES Cedric, LESCAR Stephanie, LESENECHAL Claire, LESNIAK Jerome, LESORT Sabine, LESTIN JOVER Violaine, LETEY-BABOULENE Gabrielle, LEVHA Pierre, LEVILION Juliette, LEVINE David, LEVY Alexandre, LEVY Bernard, LEVY Jonathan, LEVY Michael, LEVY Robin, LEVY-CHEMOUNI Carole, LEWI Charlotte, LEYMARIE Jean-Luc, LEYMARIE Rim, LEZMY BARACASSA Audrey, LI AH KIM Li Man Shin, LIANCE Baptiste, LIBERT Eric, LIGEN Nathalie, LIM Eric, LIM Marie-Sophie, LINNE Isabelle, LION-ALTMAYER Juliette, LIPS Jean-Christophe, LISON Nadege, LIVARTOWSKI-CARON Emilie, LO PRESTI Joanna, LOBEL Charlotte, LOCHET Nolwenn, LOKO Brice, LONG Clemence, LONGOUR Serge, LONGUEVILLE Eric, LOOTVOET Erik, LORANCE Manon, LORIGNY Severine, LOUNI PAOLI Clelia, LOUNIS Abdelkader, LOURENCO Jeremy, LOYER Camille, LOZACHMEUR Celine, LUCAS Nolwenn, LUCE Matthieu, LUCEK Annie, LUCIANI Jean-Marc, LUCK Isabelle, LUXEY Pierre-Yves, LY Chhavy, LYET Jean-Baptiste, M'SIKA-RAZON Marie, MAATOUG Gerard, MACIEIRA COELHO Lourenco, MADI-REZGUI Saskia, MAES Anne-Sophie, MAGNIER Bertrand, MAHE Michele, MAHIER Michele, MAHMOUD Sarra, MAIDENBERG Marc, MAILLE Pascal, MAITREPIERRE Isabelle, MAJER Jacques, MAJERHOLC Catherine, MALAN Sebastien, MALAPRADE Pierre, MALARMEY Eric, MALEYSSON Marie, MALFAIT Josette, MALLET Estelle, MALMARTEL Alexandre, MALVIT Christine, MAMERI Amel, MAMODALY Moise, MAMOU-YAYON Danielle, MANGOUKA Corinne, MANIKAM Diana, MANNECHEZ Daniel, MANSOURI Ali, MANSOURI Leila, MANTEAU Thierry, MAQSOOD Zubair, MARBOT Cristina, MARCAIS Marion, MARCEAUX Nadine, MARCHAL Frederique, MARECHAL Colas, MAREK Michele, MARESMA Pierre, MARGELISCH Olivier, MARIE Brigitte, MARIE Elisabeth, MARIN Jan Andi, MARINI Laurence, MARIOTON-LAVEDRINE Isabelle, MARIOTTI Marie-Gaelle, MARLIER Juline, MARROUA Rachida, MARSAN Stephanie, MARSANNE Caroline, MARTI Brigitte, MARTIN Bernard, MARTIN JABEUR Cecile, MARTIN Jean-Pierre, MARTIN MOREAU Theo, MARTIN Simon, MARTINOLE Bernard, MARTINS David, MARY Ingrid, MAS Sylvie, MASPERO Clemence, MASSE-LAFARGOUETTE Isabelle, MASSON Laurent, MASSON Nathalie, MASSY DE LA CHESNERAYE Pascale, MATHIEU Antonin, MATRONE Eric, MAUDELONDE Claire, MAURATILLE Olivier, MAUREY Christine, MAURI Daniela, MAURY Emmanuel, MAZALEYRAT Laurent, MAZEAS Yves, MAZUEL Philippe, MBESSE AMAKENE Marie Laurence, MEAS Philip, MEBAZAA Hareth, MEGHIRA Sandrine, MEGRET-FRACHON Helene, MEILLAT Isabelle, MEISTERMANN Sophie, MENANT Isabelle, MENARD Delphine, MENEGHINI Eliane, MENEGOZ Pierre-Yves, MERABET-BRAHIMI Rahima, MERCERON Olivier, MERCIER Alain, MEROT Segolene, MESGUICH Philip, MESSAS Aurel, MESTAT Pascal, METIVIER Marie, METIVIER Nathalie, MEUDEC Christel, MEUNIER Gilles, MEUNIER-CORMIER Isabelle, MEURGER Nicolas, MEURIER Francois, MEZAGUER Sabrina, MEZARD Pierre, MIALON Laurent, MICHARD Philippe, MICHAUD Valerie, MICHEL Christophe, MIDOZ Segolene, MIGEON Celine, MIGNOTTE Katell, MIGOUX Sarah, MIHOVA Stefanka, MILGRAM Sandrine, MILLOT Brigitte, MILLOT Cecile, MILLOT Pierre, MILOUDI-EDDRIEF Khedidja, MINDUS Francois, MIQUEL Francis, MITHA Gamil, MOCH Mathieu, MOGHBEL Maxime, MOIRIGNOT Pierre, MOKHTARI Mohamed, MOLIMARD Jean Marc, MOLLER Veronique, MONCHAMP Benoit, MONDO Aline, MONGIE Sandrine, MONNIER Delphine, MONTAY Daphnee, MONTEIRO-MARTIN Celine, MONTZIOLS Romain, MORALI Eric, MORAND Caroline, MOREAU Anne-Claire, MOREAU Florence, MOREL Caroline, MOREL Sebastien, MORELIERE Marie, MORIO Philippe, MORYOUSSEF-ROZENBAUM Anne, MOSCOL Adeline, MOTTEAU Sylvie, MOUAWAD Florence, MOUCHE Stephane, MOUCHEL-OUVRY Delphine, MOUILLON Jean-Pierre, MOULIN-NALTET Elisabeth, MOULYS Emilie, MOURA Bertrand, MOURAO BALSA Diana, MOURIN Matthieu, MOUSSU Lise, MOUZAOUI Mourad, MSIKA Julie, MSIKA Laurence, MUHIZI Azir, MULLER Francois, MULTON-LOGEAIS Sylvie, MULYS Denise-Laura, MUNOZ Pascale, MUSSEAU Cecile, NAAR Fabien, NABOS Axelle, NACCACHE Jean-Marc, NADJAHI Joubine, NAEGELEN Adrien, NAJEM Ilhame, NAOURI Jean-Pierre, NASREDDINE Elena, NAUDIN-ROY Adeline, NDIGO MA NDIGO NZIE Marie, NEGUIN Valerie, NEILDEZ Edouard, NEMNI Franck, NEMON Marie, NGO Natacha, NGUYEN Guillaume, NGUYEN Huu-Phuoc, NGUYEN Khac-Viet-Hung, NGUYEN Michel, NGUYEN Ngoc-Khanh, NGUYEN Patrick, NGUYEN Rosalie, NGUYEN Thi Kim Anh, NGUYEN Thi Tuyet Nga, NGUYEN VAN NHIEU Andre, NGUYEN VAN Thierry, NGUYEN-HUA Thi Thanh Huong, NGUYEN-VAN-SANG Andre, NICOLAS Violaine, NINGRE Philippe, NIVET Pascale, NIVLET Jean-Francois, NIZET Pierre, NOBILE Gaelle, NOEL Frederique, NOELE Emilie, NOUCHI-ROLLO Valerie, NOUGAIREDE Michel, NOUVIAN Christophe, NOVILLO Andres, NUGUES Sophie, NUNES Patrick, NURENI-BANAFUNZI Ismael, OHANA Benjamin, OHAYON-ELFASCI Carole, OLLIVIER Sophie, ORSINI-DEMAY Delphine, ORTHOLAN Jean-Francois, ORYE-CORLOUER Margaretha, OSTAN Pascal, OSTOIN Branko, OUACHEE Mathilde, OUGIER Evelyne, OUHIBI Lotfi, OUTREQUIN Ghislain, PAILLIEZ-SACHOT Anne, PAIRON-PENNACCHIONI Martine, PAJAK Marie-Pierre, PAME Patricia, PANDOR Isabelle, PANET Marie-Anne, PANHARD Claire, PANOT Olivier, PAOLI Charles, PARDAL RODRIGUES Paula, PARIS-HAVARD Marie-Noelle, PARISIS Philippe, PARK Sun-Young, PARLIER David, PARRA Claire, PASCAL Isabelle, PASQUET-DUMONTEIL Catherine, PATRAT Jean-Frederic, PATRELLE Blandine, PAU SAINT-MARTIN Jean-Philippe, PAUCHET Francois, PAULIN Herve, PAZAT-FROMENTIN Aline, PECILE Jean-Claude, PECQUEUR Raphael, PEETERS Joke, PELLAN Elodie, PELLIER Francis, PELOSO Camille, PELTZ-AIM Jennyfer, PEPIN Bruno, PERCIE DU SERT Alice, PEREIRA ROCHA Elsa, PEREZ Laurent, PEREZ-PEROUELLE Sophie, PERGAMENT Joel, PERIE Sophie, PERONNET-SALAUN Nathalie, PERRILLAT-AMEDE Segolene, PERRIN Thierry, PERRODIN Nathalie, PERSEEDOSS Kevin, PETIT Laurent, PETIT Stephanie, PETURAUD Cecile, PEYPOUDAT Celine, PEYRARD Lucie, PHAM Quang, PHAM THI Nhan Thuong, PHAM Van Nhat, PIBALEAU Geraldine, PICARD Agnes, PIERSON-FOUQUET Claire, PIETTE Edouard, PIEUCHON Catherine, PIGEAU Marianne, PILLON Nadja, PILLON Veronique, PINOT Juliette, PINTO Emmanuel, PINTO Jean-Jacques, PINTO-LE MAGUERESSE Sabrine, PIRES Emilia, PIROLLO Didier, PIVERT Laetitia, PIZZOCOLO Cecilia, PIZZUTI Melissa, PLAISANT Jean-Francois, PLATEK Andrzej, PLONGERON Christelle, POCHET Anne-Flore, POCHET-CARAYON Francoise, POCQUET Vincent, POINCLOUX-LE GALLO Camille, POIRIER Anne-Cecile, POIRIER LANGLOIS Isabelle, POIRSON Elodie, POITOU Caroline, POITRINE Eric, PONSIN Eric, PONTETTE Bruno, POPESCU Marius, PORTELINHA Sandra, POTIER Gregory, POTTIN Laurence, POULAIN Philippe, POUMIER Elise, POUPARD Raphaelle, POURADIER Odile, POURRAT Marie, POYET Gilbert, PRADA Yves, PRASTEAU Philippe, PRESSOUYRE Herve, PRETI Henri, PRIMARD Laurent, PRINGAULT-DESJONQUERES Maria-Pia, PROISL Olivier, PUIRAVEAU Jean-Henry, PUNG Rene, PUSSIEUX Sabine, PUYUELO CUESTA Maite, CARLI Pierre, QUEDEVILLE Fabien, QUERFANI Ouassila, QUERUEL Thomas, QUINTANEIRO Sylvie, QUINTIN Geraldine, RABANY Thierry, RABEFIRAISANA Mahalisoa, RABHI Abdelhakim, RABOURDIN Delphine, RABOURDIN Luc, RACHOU Sophie, RACINE Antoine, RAHAL Fatima, RAHMI Anne-Therese, RAIMBAULT Jerome, RAJ Srinivasan, RAMBAUD Dorothee, RAMES Alain, RAPP Nathalie, RATOVO Andrianjaka, RATOVOARISOA Manuella, RAU Laetitia, RAYBAUD Marylin, RAYSSIGUIER Anne, RAZAFINDRAZAKA Renaud, REHBINDER Marina, REIGNER-MALTRAVERSI Claire, REKKAB Ahmed, REMOND Bernard, REMY Hans-Christian, RENARD-QUISFIX Delphine, RENAUD Nicolas, RENAULT LENOBLE Alaine, RENAULT Robin, RENET Herve, RENOUF Nicolas, RETBI Isabelle, RETOURNE-NIZET Virginie, REVERDY Olivier, REVERDY-BAZIN Caroline, REY Fabrice, REY Jacques, REZAI Laurence, RIAHI Mickael, RIANT Elisabeth, RIBAT-MARCHAND Christine, RIBERRY Laurence, RICARD Guillaume, RICHARD Sylvain, RICHEMOND Michele, RICHETTA Philippe, RIEDLE Nadege, RINGOT Arthur, RIOU Olivier, RIPERT Fabienne, RIVAUD Elisabeth, RIZZI Cecilia, ROBAIN Anne, ROBERDEAU Vincent, ROBERT Camille, ROBERT Caroline, ROBERT Clara, ROBERT Francois, ROBIN Marie, ROCCA Marie-Claude, ROCCHI Delphine, RODACH Philippe, RODE Julie, RODENBOUR Charlotte, RODRIGUES Florence, RODRIGUEZ Alain, ROECKEL Eric, ROFE-SOTTO Elisabeth, ROGER Stephane, ROGOWSKA Karolina, ROISNE-LEFEVRE Sophie, ROJAS Miguel, ROLLAND Julien, ROMAN Alexandre, ROMESTAN Angelina, ROMMELAERE Caroline, RONIN Sara, RONNE Florence, ROSA Manuela, ROSENCHER Julien, ROSILIO Myriam, ROSS Cecile, ROSSET Thierry, ROSSI Jean-Charles, ROSSIGNOL Sophie, ROTHAN Gabriel, ROUCOULES Brice, ROUDEIX Remi, ROUDIL Franck, ROUILLEAULT Alice, ROULLIER Benoit, ROULLIER Vanessa, ROUMIEU Sigolene, ROUSSEAU Isabelle, ROUSSEAU Martine, ROUSSEL Jeremy, ROUSSELLE Maxime, ROUSSI-BARGAS Monique, ROUYER Marie-Louise, ROZE Mathilde, ROZENBLAT Marc, ROZENBLAT-ZERBIB Sabine, ROZENBLUM Eric, ROZENFELD Richard, ROZMAN Marc, RUSSO Patrick, SAAB Jade, SAADA Arnaud, SAADA Benjamin, SAADA Sandrine, SABAH-MENEGHEL Coralie, SABER Samir, SACRABANY Sylviane-Marie, SADAT Isabelle, SAGOT Pascal, SAID IBRAHIM Toutah, SAINT GEORGES Beatrice, SAINT-DENIS Penelope, SAINT-ETIENNE Julien, SAINT-FELIX Coraline, SAINTE-ROSE Melanie, SAINTHERANT Aude, SAIOVICI David, SAISSET Pierre, SALATO Philippe, SALAUN Bernard, SALES Fanny, SAMAIN Clementine, SAMAMA Ruth, SANTINI Arlette, SARAZIN Veronique, SARDA Carine, SARFATI Herve, SARFATI Marc, SARLANDIE DE LA ROBERTIE Emeline, SAROTTE Richard, SARRON Tulio, SATCHIVI-LUSUASU Rose Marie, SAUNAL Valerie, SAUPIN-TRICAUD Veronique, SAUSSEREAU Lucile, SAUVAGE-RIGAL Sophie, SAVALE Camille, SAYNAC Yohan, SCEMAMA Corinne, SCEMAMA Guy, SCEUTENAIRE Wilfried, SCHALLER Isabelle, SCHILDT Pauline, SCHLEPP Emmanuelle, SCHMIDT Carole, SCHMITT-TRIN Florence, SCHNELL Laura, SCHULLER Marie-Pascale, SCHWARTZ Jean-Claude, SCHWARTZ Yves, SCOLARY Francoise, SEANG Sophie, SEAUX Juliette, SEBAN Alain, SEBAOUN Gilles, SEBBAG Pierre, SEBBAN Katia, SEBBAN-BOUKHRIS Esther, SEBBAN-ZANA Chloe, SEBILLON Elise, SEE Jacques, SEIBOLD Matthieu, SEIF Aline, SELLEM HABIB Elodie, SELLERIER Anne Marie, SELLIER-VICENTE Anne-Sophie, SELLOUK Jeremy, SEMERCIYAN Armand, SENE Emmanuel, SENIOR Jacques, SENN Clotilde, SENTENAC Laure, SEREY Patrick, SEREY Regis, SERGOT Ewa, SERRANO Emanuelle, SERRANO Stephane, SERRE Jean-Louis, SERS-PERRIN Helene, SESTIER Valerie, SFAR-KHATOUNIAN Catherine, SHIN Robert, SIDORKIEWICZ Stephanie, SILBER Jeremy, SILVERA Eric, SIMEON Yves, SIMMET Marion, SIMON Eric, SIMON Thomas, SIMONET Philippe, SIMSEK Nurcan, SIN Chou Ka Ying, SITBON Katia, SIVALINGAM Vennila, SIVARASALINGAM Kajajini, SIXOU Pierre, SOBIAK Olivier, SOHIER-ATTIAS Julie, SOKOLSKI Denis, SOLMAZ-YAVULU Meryem, SONG MATHA David, SONTAG Christelle, SORRE Amandine, SORRIAUX Claire, SOUBELET Philippe, SOUDRY-MILLE Colette, SOUFACHE Elodie, SOURZAC Robert, SOUSSAN Robert, SOUTIRAS Frederique, SOUVERVILLE Claire, SOYEZ Delphine, SPAGNOL Anne-Laure, SPECIEL Philippe, SROUSSI Deborah, STAN Corina Denis, STANCE Corinne, STAVRAKIS Kosta, STEINEBACH Alice, STERN Benedicte, STOCLIN Juliette, STOYANOVA Miglena, SULTAN-AMAR Valentine, SUSSAN Juliette, SYLLA Siraba, SZILVASSY Peter, TAGLIAMONTE Florence, TAIEB Jeremy, TAKALI Zied, TAKLA Patrick, TALBI Chaima, TALHOUARN Vanessa, TAN David, TANCHON Vanessa, TANFEUDEU Kevin, TARAKI Kevin, TARANDO Alain, TARDIEU Andre, TARDY Carole, TARGOWLA Nathalie, TASSEL Clement, TAUPIN Guillaume, TAWIL Sophie, TAZI Zeineb, TEBBANI Nadia, TEBOUL Francois, TEBOUL Patrick, TEILLET Marc, TELLE Pauline, TEMAN Corinne, TERMINET Alain, TERNISIEN Isabelle, TERNON Rodolphe, TERRIER Marion, TESSIER Aude, TESSIER Yannick, THANACODY Franck, THEVENON Christophe, THIBAUD Lucile, THIEBAUT Luc, THIERRY Brigitte, THIRION Florence, THIRION-MONFORT Marie-Annick, THOMAZEAU Maximin, THONG-BUISSON Amelie, TILLIARD Sylvie, TINDO Ngoumo Lydie, TIRILLY Caroline, TIROUCHE Sofiane, TISSIER Nathalie, TMIM Eve, TOBELEM Elyane, TOBELEM Robert, TOLEDANO Prosper, TOLLEMER Beatrice, TONION Sophie, TORCHIN Danielle, TORTIGET Delphine, TOUALBI Amel, TOUPOINT Pierre, TOURNIER Gilles, TOURNIER-SOBIAK Anne-Dominique, TRABELSI Sarah, TRAINOY Pascal, TRAJANOV Karolina, TRAN Nha Huong, TRAN QUANG Binh, TRANG Emilie, TRARIEUX Fabrice, TRICON Christophe, TRINCARD Blandine, TRINQUET Pierre, TROME Daphne, TRUONG Camille, TRUONG Phuong Anh, TUMSON Sylvaine, TUR Emilie, TURGIS Vincent, URBAIN Frederic, UTHURRIAGUE Sylvie, UZAN Julien, UZAN Pierre, UZAN Sandy, UZAN-FEDIDA Johanna, UZUREAU Didier, VACHER Sandrine, VADOT Camille, VALENDOFF Joel, VALENTIN Cecile, VALLADAS Francoise, VALLETEAU Anne Sophie, VALMALLE Nicolas, VAN BATTEN Pascal, VAN DEN BROUCKE Julie, VAN-HOUTTE Laetitia, VANDENDRIESSCHE Juliette, VANPOPERINGHE Nicolas, VANTHOMME Benedicte, VASSEUR Frederique, VASSEUR Geraldine, VASSEUR Marc, VATIN Severine, VAUTHEROT-CHOMIAK Adeline, VAUTHIER Marc-Andre, VENNEGUES Annick, VENTRE Clara, VENTURI Cecile, VERGERON Jean, VERGIER Romain, VERGNE Julien, VERGNE Leonore, VERJANS Jean-Luc, VERRECCHIA Stephanie, VERRIERE Camille, VERSTRAETEN Jean-Claude, VETILLARD Pauline, VETTERL Mathilde, VIARD Danielle, VICTOROFF Catherine, VIEILLE Cecile, VIEIRA Patricia, VIENNE Audrey, VIGNE Matthieu, VILLANUEVA Christine, VILLARET Christian, VILLIERS-MORIAME Michel, VINCENDON Enora, VINCENSINI Jean-Paul, VINCENT Valerie, VINCENT Victor, VIOMESNIL Noemie, VIOMESNIL Vanessa, VITTE Caroline, VO-LIEN-HA Patrick, VOIRIN Stephane, WALLET Jean-Pierre, WALSER Sebastien, WALTER Sylvie, WANSI TCHUINGOUA Mathias, WATREMEZ Claire, WAZANA Delphine, WEEGER Benoit, WEINBERG Eric, WEIS Jean-Christophe, WEIZMANN Ledicia, WEYL Marine, WICART-POQUE Fabienne, WILLEMIN Jean-Pierre, WINTER Francoise, WOLF Sylvie, WORMSER Laurent, YACOUBOVITCH Jacques, YANA Philippe, YOUDI Lili, YOUSSEFIAN Anne, ZABAR Jehan, ZAHZAM Lucie, ZAIED Melissa, ZAIM Nora, ZAKI Stephanie, ZARKA Loraine, ZEHOU Soulef, ZENATTI Sonia, ZENOU Laurence, ZERBIB Celine, ZERBIB David, ZERR Philippe, ZERT-KAMOUN Marie-Anne, ZIDI Erwan, ZIMOLO Anne-Marie, ZING Emmanuel, ZOU AL GUYNA Line, ZOUARH Nadia, ZOUARI Morched, ZRIHEN Henri, ZUILI-BITBOL Myriam, ZYSMAN Claire
